# Agents Targeting the Bacterial Cell Wall as Tools to Combat Gram-Positive Pathogens

**DOI:** 10.3390/molecules29174065

**Published:** 2024-08-27

**Authors:** Aliaksandr Zhydzetski, Zuzanna Głowacka-Grzyb, Michal Bukowski, Tomasz Żądło, Emilia Bonar, Benedykt Władyka

**Affiliations:** 1 Department of Analytical Biochemistry, Faculty of Biochemistry, Biophysics and Biotechnology, Jagiellonian University, Gronostajowa St. 7, 30-348 Cracow, Poland; aliaksandr.zhydzetski@uj.edu.pl (A.Z.); zuzanna.glowacka-grzyb@doctoral.uj.edu.pl (Z.G.-G.); m.bukowski@uj.edu.pl (M.B.); tomasz.zadlo@doctoral.uj.edu.pl (T.Ż.); emilia.bonar@uj.edu.pl (E.B.); 2 Doctoral School of Exact and Natural Sciences, Jagiellonian University, Prof. St. Łojasiewicza St. 11, 30-348 Cracow, Poland

**Keywords:** peptidoglycan, bacterial cell wall, antibiotic, endolysin, lysostaphin, *Staphylococcus aureus*, Gram-positive bacteria, peptidoglycan hydrolase

## Abstract

The cell wall is an indispensable element of bacterial cells and a long-known target of many antibiotics. Penicillin, the first discovered beta-lactam antibiotic inhibiting the synthesis of cell walls, was successfully used to cure many bacterial infections. Unfortunately, pathogens eventually developed resistance to it. This started an arms race, and while novel beta-lactams, either natural or (semi)synthetic, were discovered, soon upon their application, bacteria were developing resistance. Currently, we are facing the threat of losing the race since more and more multidrug-resistant (MDR) pathogens are emerging. Therefore, there is an urgent need for developing novel approaches to combat MDR bacteria. The cell wall is a reasonable candidate for a target as it differentiates not only bacterial and human cells but also has a specific composition unique to various groups of bacteria. This ensures the safety and specificity of novel antibacterial agents that target this structure. Due to the shortage of low-molecular-weight candidates for novel antibiotics, attention was focused on peptides and proteins that possess antibacterial activity. Here, we describe proteinaceous agents of various origins that target bacterial cell wall, including bacteriocins and phage and bacterial lysins, as alternatives to classic antibiotic candidates for antimicrobial drugs. Moreover, advancements in protein chemistry and engineering currently allow for the production of stable, specific, and effective drugs. Finally, we introduce the concept of selective targeting of dangerous pathogens, exemplified by staphylococci, by agents specifically disrupting their cell walls.

## 1. Introduction

**Figure 1:**
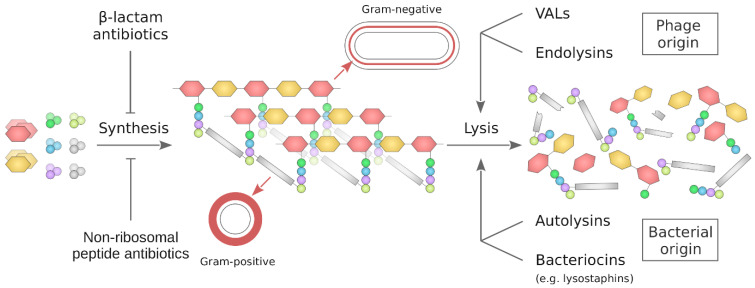
Antimicrobial agents acting on the bacterial cell wall. Classic antibiotics, low-molecular-weight compounds, and non-ribosomal peptides interfere with peptidoglycan synthesis, which is the main building material of the bacterial cell wall. The peptidoglycan layer in Gram-positive bacteria is thick and exposed to the outer environment. In contrast, in Gram-negative bacteria, the peptidoglycan layer is considerably thinner and covered by an outer membrane, which limits the penetration of antimicrobial agents, particularly those with high molecular weight. High-molecular-weight agents are proteinaceous and may be divided into two groups: lysins of phage origin (virion-associated lysins [VALs] and endolysins) and enzymes of bacterial origin (autolysins and bacteriocins). These antimicrobial agents are responsible for the enzymatic lysis of the bacterial cell wall.

In May 2024, the WHO published the updated version of the Bacterial Priority Pathogen List, containing 24 drug-resistant pathogens. Among them are Gram-positive bacteria, such as *Staphylococcus aureus*, *Enterococcus faecium*, and *Streptococci*, that are resistant to cell-wall-targeting antibiotics. Moreover, many Gram-negative pathogens resistant to drugs affecting peptidoglycan layer synthesis are of the highest threat to public health [1]. This creates an urgent need for novel antibacterial agents against these drug-resistant pathogens. On the other hand, it points to the bacterial cell wall, especially the peptidoglycan, as a target for many antibiotics currently used for the treatment of drug-sensitive pathogens. Although bacteria are able to develop resistance to this type of antibiotics, the cell envelope is still one of the primary targets for novel antimicrobials for many reasons. Firstly, the cell wall is not present in animal and human cells, which argues for the safety of drugs targeting this structure for the infected host. Moreover, differences in the structure of the cell wall, primarily exemplified by Gram-positive and Gram-negative bacteria, enable selective targeting within these two groups (Figure 1). Furthermore, in the case of Gram-positive bacteria, the thick peptidoglycan layer differs in the composition of the peptide part among genera, species, and even strains, which provides a basis for designing even more specific antibacterials. This is of special importance for at least two reasons. First, the complexity of the microbiota of the host is tremendous. Dozens of various bacteria reside not only in the gastrointestinal tract but also on the skin and in the urogenital system. Dysbiosis, induced by a broad range of antimicrobials used to treat infection, may have serious negative consequences for the host. Secondly, the application of such antibiotics puts pressure on the whole microbiota to develop resistance, which may be further spread by horizontal gene transfer. Therefore, high specificity should be a key factor in the development of new antibacterial agents. Here, we review agents acting on the bacterial cell wall, both the widely used antibiotics as well as other high-molecular-weight agents of peptide and protein nature. Historically, progress in antibiotic discovery, modifications, and refinements has reflected a constant arms race in which we are currently facing multidrug-resistant pathogens with, at best, very limited treatment options. Therefore, the use of alternative agents, such as bacteriocins, autolysins, and phage lysins, is increasingly being considered to overcome the shortage of new antibiotics and the already developed resistance mechanisms. Moreover, we discuss the concept of microbiota-friendly antibacterials, exemplified by the lysostaphin variant specifically targeting *S. aureus*.

## 2. Structure, Synthesis, and Remodeling of Peptidoglycan

Peptidoglycan (PG), also known as murein, is a crucial element of the bacterial cell wall. Although the morphology of the cell wall is a major factor dividing bacteria into Gram-positive (monoderm) and Gram-negative (diderm) ones, the molecular structure and synthesis of PG are similar. The main difference comes from the thickness of the PG layer and its localization within the cell envelope. Gram-positive bacteria have a thick (30–100 nm) PG multilayer surrounding the cell membrane, which forms a protective shell [2]. Gram-negative bacteria are surrounded by a thin (<10 nm) PG layer, which is itself surrounded by an outer membrane containing lipopolysaccharide.

**Figure 2:**
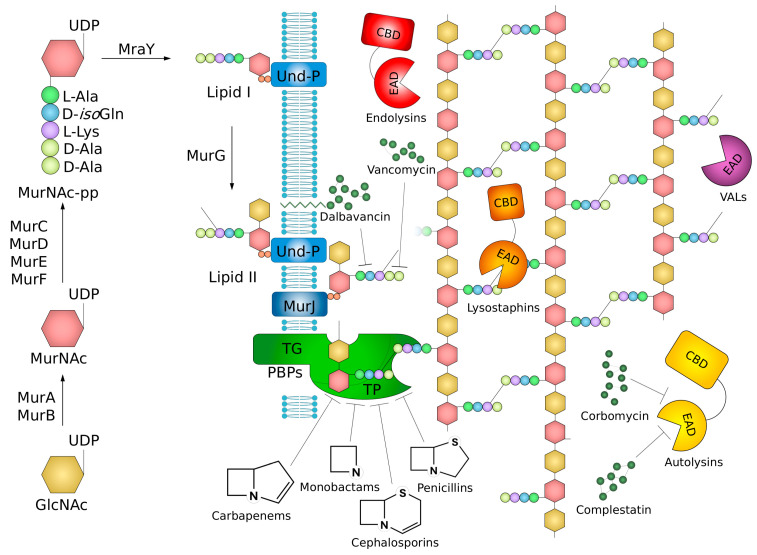
Biosynthesis and disassembly of peptidoglycan (PG). The synthesis of PG begins in the cytoplasm, where GlcNAc–UDP is transformed into MurNAc–UDP by MurA and MurB enzymes. It is followed by a stepwise addition of five amino acids (the stem peptide) by MurC, MurD, MurE, and MurF. MurNAc–pentapeptide (MurNAc–pp) is anchored by two phosphate molecules to membrane-bound undecaprenyl lipid carrier (Und–P), creating Lipid I. MurG then adds GlcNAc to MurNAc–pp, which together form Lipid II. FemA, FemB, and FemX enzymes add an interpeptide bridge to the third amino acid in the stem peptide. The finished monomer is translocated to the outer edge of the membrane by MurJ flippase, where penicillin-binding proteins (PBPs) perform binding of Lipid II to the previous glycan strand by the transglycosylase (TG) domain. The transpeptidase (TP) domain crosslinks peptidoglycan by joining strands with interpeptide bridges. The activity of PBPs is inhibited by conventional antibiotics (penicillin, carbapenems, monobactams, cephalosporins). Acquisition of resistance results in mutated forms of PBPs; therefore, another approach is the use of glycopeptides such as vancomycin and dalbavancin (which interact with the stem peptide, preventing transglycosylation) or corbomycin and complestatin (which interfere with autolysins by preventing PG remodeling). Antibiotics and glycopeptides interfere with PG biosynthesis, whereas endolysins and lysostaphins activities affect mature cell walls.

PG is composed of linear strands of repeating disaccharide units cross-linked by short peptide side chains (Figure 2). The disaccharide subunit is conserved and consists of N-acetylglucosamine (GlcNAc) and N-acetylmuramic acid (MurNAc) residues linked by β-1,4 glycosidic bonds [3]. The unique bacterial sugar MurNAc in the glycan strand is bonded to the N-terminus of a five-amino-acid linear peptide (stem-peptide) via an amide bond. The first L-alanine of this pentapeptide is typically followed by D-isoglutamine. In the third position is either L-lysine (in *S. aureus, S. pneumoniae*, *E. faecalis*, and *E. faecium*) or meso-diaminopimelate (m-DAP) (in *B. subtilis*), followed by the dipeptide D-Ala-D-Ala. Typically, the ε-amino group of L-Lys is bound to an interpeptide bridge (branching/cross-linking peptide) ranging from one to seven amino acid residues. In the case of *S. aureus*, the cross-linking peptide is pentaglycine, in *S. pneumoniae*, it is the dipeptide L-Ala-L-Ser or L-Ala-L-Ala, whereas in *E. faecalis*, it is L-Ala-L-Ala. The cross-linking of individual glycan strands generally occurs via the formation of an amide bond between the L-Lys/m-DAP-attached cross-linking peptide and the backbone carbonyl of the fourth amino acid of another stem peptide, with the loss of the terminal D-Ala [4].

PG biosynthesis takes place in several stages (Figure 2). The first steps start in the cytoplasm with the transformation of UDP–GlcNAc to UDP–MurNAc, followed by the stepwise addition of amino acids, resulting in the UDP–MurNAc–pentapeptide. These reactions are catalyzed by MurA–F enzymes. Next, these stages are followed by the assembly of the phosphor–MurNAc pentapeptide with a membrane-embedded undecaprenyl phosphate lipid carrier (Und–P) to produce a lipid-linked monosaccharide intermediate known as Lipid I, which is then glycosylated with GlcNAc to form the disaccharide compound, namely Lipid II. Then, amino acids of the cross-bridge peptide, if present, are added to the third position of the pentapeptide stem-chain, and Lipid II is translocated across the membrane by flippase MurJ. At the final step of PG biosynthesis, Lipid II monomers are polymerized outside the cell. This process requires transglycosylation and transpeptidation, which are catalyzed by enzymes commonly referred to as penicillin-binding proteins (PBPs). The enzymes may have peptidoglycan glycosyltransferase or transpeptidase activity or be bifunctional. The Und–P lipid carrier released during glycan chain polymerization is then recycled back to the inner side of the membrane by the UndP flippases UptA and/or PopT to enable another round of precursor synthesis [5,6,7].

At the same time, the cell requires the mechanisms to allow it to rearrange and reassemble synthesized mature PG to provide cellular expansion during the cell cycle. These processes are controlled by a group of enzymes termed autolysins, including glycan hydrolases (degrading within the glycan strands), amidases (digesting the amide bond between MurNAc and the stem peptide), endopeptidases (cleaving the peptide bond in crosslinking or the stem peptide), and carboxypeptidases (cutting off the terminal residues of the stem peptide). The combined action of autolysins creates space in the covalently closed mesh of the peptidoglycan sacculus for the incorporation of newly synthesized peptidoglycan chains, which is an indispensable element of cell wall remodeling and turnover (Figure 2). Specificity and peculiarity in the case of PG expansion lie in the fact that crosslink-specific endopeptidases are essential for the expansion of the thin PG layer of Gram-negative bacteria, whereas monoderms expand their thick murein layer by cleaving within the stem peptide. Thus, successful cell wall assembly is dependent on the cooperation of both the synthesis and hydrolysis processes [3,8].

Because PG plays a fundamental and essential role in the bacterial life cycle, the enzymes involved in its synthesis, remodeling, and repair are highly conserved and may serve not only as primary targets of antibacterial agents but also represent possible new antibacterial agents in themselves.

## 3. Agents Targeting Peptidoglycan Synthesis

Cell wall biosynthesis is a complex process with multiple steps involving many proteins, each of which can be a target for antibiotics. Inhibitors of cell wall synthesis are one of the most effective and extensively used classes of antibiotics; among them are β-lactams and glycopeptides.

### 3.1. β-Lactams and Mechanisms of Resistance to Them

β-lactams comprise a group of antibiotics that contain a beta-lactam ring, consisting of three carbon atoms and one nitrogen atom, in their chemical structure. They are further divided into penicillins, cephalosporins, monobactams, and carbapenems. In response to exposure to β-lactams, bacteria have developed a number of mechanisms to avoid their harmful effects. β-lactamase-based resistance, altered penicillin-binding proteins (PBPs), and resistance mediated by impermeability or efflux are among them.

#### 3.1.1. Penicillins

**Figure 3:**
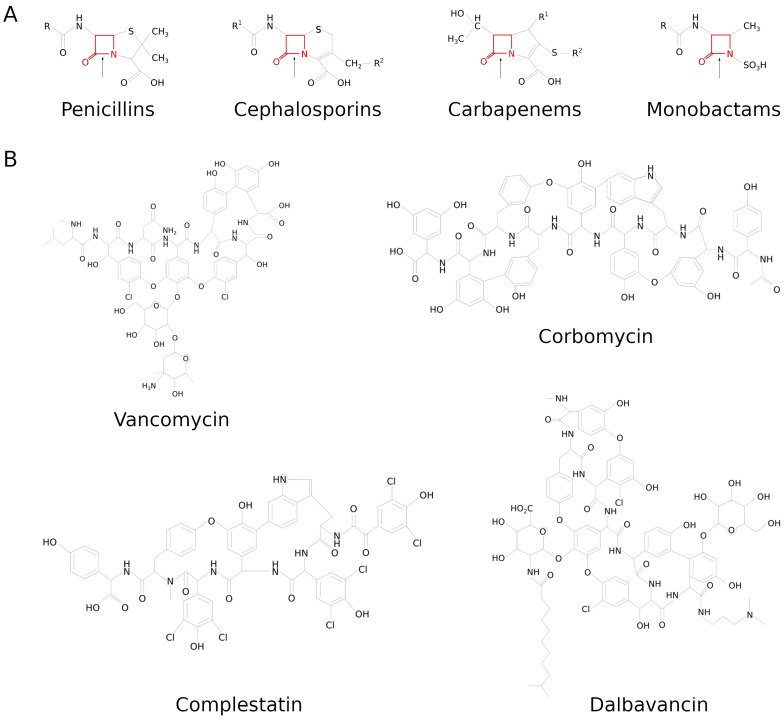
The structural formulas of the cell-wall-targeting antibiotics. (**A**) The structures of the cores of β-lactam antibiotics. The β-lactam ring is colored in red. The bond hydrolyzed by β-lactamases is marked with an arrow. R, R1, and R2, variable functional groups. (**B**) The structural formulas of exemplary glycopeptide antibiotics.

Penicillins are the oldest discovered class of antibiotics, with the most notable representative being penicillin [9]. Here, the β-lactam ring is fused to a five-member thiazolidine ring (Figure 3A). This class contains both natural (e.g., benzopenicillin) and semisynthetic (e.g., methicillin and ampicillin) members. Methicillin was created to target penicillin-resistant staphylococci, yet within the first year of its introduction, the first methicillin-resistant *S. aureus* (MRSA) strains were identified [10]. The development of resistance against other semisynthetic penicillins, such as oxacillin, cloxacillin, and flucloxacillin, followed suit. Aminopenicillins were introduced as a third generation of compounds related to original penicillin, with the advantage of a broader spectrum of activity and decreased susceptibility to β-lactamases [11], enzymes opening the β-lactam ring. New penicillins consist of a chemically modified core molecule, 6-aminopenicillanic acid, leading to the creation of imine derivatives [12,13] that exhibit antibacterial activity more potent than that of ampicillin.

#### 3.1.2. Cephalosporins

In cephalosporins, the β-lactam moiety is fused to a six-member dihydrothiazine ring (Figure 3A). Such a structure allows for multiple modifications of C3 and C7 carbons with various side chains, enhancing the antibacterial activities of those antibiotics [14]. Cephalosporins are divided into generations according to their respective spectrum of recognized bacteria, although they still remain an alternative to penicillins in mild to severe infections [15]. The first discovered and most widely used cephalosporin is cephalotin. Cephalotin and other first-generation cephalosporins primarily work against Gram-positive cocci, including methicillin-sensitive *S. aureus* (MSSA). The next generations of cephalosporins were successively developed in order to overcome growing resistance from previous generations. The newest fifth generation is represented by ceftaroline, which exhibits a broad spectrum of activity, covering both Gram-positive and Gram-negative bacteria, and has been proven to be effective in the clearance of MRSA [16,17]. In these bacteria, PBP2a is mutated to shield the active site from access by the majority of β-lactams. Only after PG binding does PBP2a undergo conformational changes, potentiating access to the active site. Ceftaroline possesses a side chain mimicking peptidoglycan structure, which can activate PBP2a and allow binding to the active site [18] and its inhibition. Two molecules of ceftaroline are needed for successful inhibition of PBP2a: one molecule binds to the allosteric site, triggering a change in conformation and permitting another molecule to enter the active site, creating a stable acyl–enzyme complex (Protein Data Bank [PDB] ID: 3ZFZ) [19]. Ceftobiprole is another fifth-generation cephalosporine exhibiting extremely high affinity towards the PBP2a-active site, creating a stable complex that slowly undergoes hydrolysis. Contrary to ceftaroline, ceftobiprole interacts with all staphylococcal PBPs [20], which causes its excellent activity against these bacteria. Moreover, ceftobiprole also binds PBPs in *E. faecalis* as well as many Gram-negative pathogens, which significantly broadens its antibacterial spectrum [21].

#### 3.1.3. Carbapenems

Carbapenems are highly versatile antibiotics, able to kill a broad spectrum of bacteria regardless of their resistance profile. The β-lactam core of carbapenems is fused to a pyrroline (penem) ring, which undergoes tautomerization to form an imine highly resistant to hydrolysis (Figure 3A) [22]. Moreover, a characteristic conformation of the side chain in a *trans* position instead of a regular *cis* in other β-lactams results in decreased sensitivity of carbapenems to β-lactamases. Since they are one of the most effective compounds in infection treatment, carbapenems are used only as a last-resort drug in common infections [23]. Carbapenems disrupt cell wall synthesis via transpeptidation inhibition, and their principal targets are PBPs (1a, 1b, 2, and 3). The proteins differ between Gram-positive and Gram-negative bacteria, which have a reflection in their affinity to carbapenems. Meropenem binds PBP2 in *E. coli*, while in *S. aureus,* PBP1 and PBP4 are also recognized, which makes it an efficient antibacterial drug [24]. Current trends [25] in carbapenem research focus more on seeking mixtures with β-lactamase inhibitors than the development of novel molecules. Such combinations expand the effective range of each compound, resulting in the successful elimination of highly resistant pathogens. The approach was effective in treating infections where the application of other antibiotics was limited or impossible [26,27].

#### 3.1.4. Monobactams

Contrary to other β-lactams, the central ring of monobactams is not fused to another heterocycle ring structure (Figure 3A). This unique feature makes monobactams more stable to hydrolysis by β-lactamases [28]. Monobactams, exemplified by the first and only admitted aztreonam, interfere with cell wall synthesis by forming covalent acyl–enzyme intermediates with PBP3. Therefore, these antibiotics are primarily used as antibacterial agents against Gram-negative pathogens, including *Pseudomonas aeruginosa* [29]. In the face of increasing resistance to aztreonam, new monobactam derivatives were synthesized. Among the novel synthesized compounds, there are AIC499 [30] and LYS288 [31], as well as monobactam sulfonates [32] 0073 and IMBZ18g [33] exhibiting resistance to extended-spectrum β-lactamases. Moreover, in the newest monobactams, “the Trojan horse” strategy was applied by conjugating aztreonam with a siderophore moiety, allowing the antibiotic to enter the cell via regular iron uptake systems [34].

### 3.2. β-Lactamase Based Resistance

β-lactamases are hydrolytic enzymes that recognize the β-lactam ring and degrade the amide bond in the center of the compound. The β-lactamase-based resistance appeared soon after the introduction of penicillin for wide use. The number of those enzymes steadily increases in response to the application of next-generation β-lactams [35]. This wide range of β-lactamases can be divided into many categories based on functional properties, affinity towards their targets, and response to inhibitors. According to similarities in amino acid sequence and exhibited activities, β-lactamases can be organized into four classes. Classes A, C, and D cover proteins with serine residue at their active site (serine-β-lactamases, SBLs), but with no significant structural similarities between the classes, while class B contains metallo-β-lactamases (MBLs), requiring a zinc cofactor for activity [36]. Class A represents a very diverse family of proteins; therefore, it contains several subclasses [37]. Usually, they are chromosomally encoded and highly prevalent in clinical isolates. Among them, there are well-known penicillinases, but also cephalosporinases and carbapenemases—one of the most dangerous β-lactamases due to the limited or lack of available inhibitors [38]. Class C [39], otherwise known as AmpC-type, covers multiple β-lactamases expressed mainly by Gram-negative bacteria. It also contains cephalosporinases, hence the historical name of the class [40]. Class D-lactamases were of less concern, until the emergence of plasmids containing class D-carbapenemases, oxacilinases OXA-23, and OXA-146 [41], with predominant occurrence in *Pseudomonas aeruginosa* [42] and subsequent transmission to *Acinetobacter baumanii*. OXA enzymes differ in activity, which may be restricted to penicillins and the first generation of cephalosporins or may have an extended spectrum to late-generation cephalosporins and even carbapenems [42]. Extended-spectrum β-lactamases (ESBLs) are also in class A. They are spread by horizontal gene transfer together with resistance genes to other classes of antibiotics, such as aminoglycosides, chloramphenicol, sulfonamides, and tetracyclines. Therefore, antibiotic options for the treatment of ESBL-producing organisms are extremely limited. Class B-lactamases (MBLs) differ significantly from SBLs since they rely on zinc cofactor to exhibit their activity. MBLs do not interact with substrates unless two zinc ions are bound to them. Substitution of zinc with other cations results in diverse substrate binding and affects catalysis [43]. Originally, they were chromosome-encoded, but plasmids carrying genes for carbapenemases–MBLs have been identified since 1990 [44]. To prevent the spread of antibiotic resistance via β-lactamases, various inhibitors are researched. The first discovered β-lactamase inhibitor was clavulanic acid, which irreversibly binds serine residue in the active site of many representants of class A-lactamases. To further enhance their efficacy, such inhibitors are used in combinations with β-lactam antibiotics, e.g., amoxicillin/clavulanate or ampicillin/sulbactam [45]. Currently, the most promising results relate to the use of avibactam combinations. In comparison to other clinically approved inhibitors [46], avibactam is structurally distinct since it does not contain a β-lactam ring. In addition, it has an unusual mechanism of inhibition. While the covalent inhibition proceeds in a similar manner via the opening of the avibactam ring, the reaction is reversible, whereby deacylation results in regeneration of the intact compound as opposed to hydrolysis and turnover of β-lactam core inhibitors (PDB ID: 4OOY) [47].

### 3.3. Penicillin-Binding Proteins (PBPs)

Penicillin-binding proteins (PBPs) are involved in the biosynthesis of peptidoglycan and are targets for β-lactam antibiotics. Bacteria possess varying numbers and types of PBPs, both essential and nonessential for their growth. Additionally, their different affinities for β-lactams affect the sensitivity of bacteria to these antibiotics. Proteins of the PBP family exhibit various activities, such as binding the elements (MurNAc and GlcNAc) of cell wall monomers and the assembly and crosslinking of peptidoglycan strands [48]. PBPs are classified by their enzymatic activity into three classes: class A, which gathers bifunctional PBPs with both glycosyltransferase and transpeptidase activities; class B, encompassing transpeptidases; and class C, which contains carboxypeptidases and endopeptidases [48]. If the binding of β-lactams to PBP does not disturb cell proliferation or cause cell death, these proteins are called low-affinity PBPs. *S. aureus* possesses four PBPs, namely PBP1, PBP3, and PBP4, which are transpeptidases, and PBP2, which is a bifunctional transpeptidase–transglycolase, all efficiently targeted by β-lactams. In contrast, MRSA has an additional transpeptidase, PBP2a, encoded by the *mecA* gene of staphylococcal cassette chromosome mec (SCCmec) [49]. The protein exhibits decreased affinity towards β-lactams, allowing *S. aureus* to continue peptidoglycan synthesis despite the presence of antibiotics. The crystal structure of PBP2s revealed a closed active site, which prevents the interaction with β-lactams but also peptidoglycan monomers. However, PBP2a also has an allosteric site, which, when occupied, opens the active site to permit substrate entry. Currently known allosteric ligands are peptidoglycan monomers (PDB ID: 3ZG5) [50]. β-lactam-resistant enterococci possess five genes encoding PBSs. While PBP1 and PBP2 are not essential for bacterial survival in the presence of antibiotics, a lack of PBP4 in *E. faecalis* and PBP5 in *E. faecium* leads to penicillin hypersensitivity [51]. In contrast to staphylococcal PBP2a, enterococcal PBP4 and 5 lack an allosteric site. However, the apparent low affinity for β-lactams is conferred not by the weak binding of the inhibitor but by the structure and dynamics of the active site, which facilitates the acyl–enzyme inhibitor complex hydrolysis, increasing β-lactam antibiotic turnover.

### 3.4. Resistance Mediated by Impermeability or Efflux

The third major mechanism in β-lactam resistance relies on the prevention of uptake or actively decreasing the amount of antibiotics inside the cell. The mechanism is limited to Gram-negative bacteria, where the PG layer is encircled by the outer membrane. A reduction in sensitivity to β-lactam antibiotic may result from the number and structure of outer-membrane transporters, porins, and efflux pumps, which up-take and pump out the drugs, respectively. Porins are trimeric channel proteins that enable the exchange of small polar molecules. Mutations in porins contribute to the development of antibiotic-resistant phenotypes. For example, a single-point mutation of PorB in *Neisseria meningitidis* reduces its size and alters its electrostatic properties, which results in reduced permeability for β-lactams [52]. Moreover, the upregulation of efflux pumps has been reported in clinical isolates of drug-resistant Gram-negative pathogens [53]. In *P. aeruginosa*, resistance was obtained due to ubiquitous mutations in the repressors of twelve efflux pumps [54]. Similar types of efflux pumps exist in *Enterobacter* spp. The AcrAB–TolC tripartite efflux system is responsible for the most efficient intrinsic and acquired resistance.

### 3.5. Glycopeptide Antibiotics

β-lactams are antibiotics of first choice, but patients with serious infections, those allergic to β-lactams or those infected with β-lactam-resistant pathogens may be treated with other inhibitors of peptidoglycan (PG) synthesis. Glycopeptide antibiotics (GPAs) are non-ribosomal glycosylated tricyclic or tetracyclic heptapeptides that may have additional lipophilic fatty acid side chains (Figure 3B) [55]. GPAs are large complex molecules containing functional groups (amino, carboxyl, hydroxyl) that enable their modification in order to overcome bacterial resistance [56,57]. GPAs inhibit the synthesis of bacterial cell walls by binding to their molecular target, the *C*-terminal of D-Ala-D-Ala terminus of the PG precursors (Lipid II), exposed on the external surface of the cytoplasmic membrane. This prevents the substrate from being subjected to transpeptidation and transglycosylation reactions by PBPs, resulting in inhibition of PG synthesis or weakening of its cross-linking, which destabilizes the cell wall integrity and makes the bacteria susceptible to lysis [57]. GPAs are effective against life-threatening infections caused by Gram-positive pathogens, particularly against clinical isolates of the *Staphylococcus*, *Streptococcus*, and *Enterococcus* genera, including drug-resistant strains, such as the intestinal anaerobe *Clostridioides difficile* [57]. The core of first-generation GPAs includes structurally closely related vancomycin and teicoplanin. The former was discovered in the 1950s and approved for the treatment of bacterial infections in 1958 [55,58], whereas teicoplanin was first reported in 1978 and approved in Europe in 1998, currently used in many countries but not in the United States [59]. The distinctiveness of these antibiotics lies in the lack of cross-resistance with other classes of antibacterial drugs, the significant delay between their discovery and the emergence of resistance, and their activity against penicillin-resistant Gram-positive bacteria [59]. Teicoplanin, compared to vancomycin, is additionally acylated with a fatty acid chain, which makes the molecule more acidic and lipophilic. This results in an increased half-life in serum and better tissue penetration, which improves pharmacokinetics and thus antimicrobial potency. Indeed, teicoplanin combined with conventional vancomycin therapy gave better results in the treatment of pulmonary infections caused by methicillin-resistant staphylococci [60]. Targeting the major building block (Lipid II) of PG with vancomycin makes it extremely difficult to develop resistance to GPAs. However, the extensive use of first-generation GPAs has resulted in the transfer of resistance genes from bacteria (actinomycetes) naturally resistant to GPAs, first to enterococci and then to staphylococci. The set of genes is responsible for the synthesis of a modified variant of Lipid II, in which the susceptible target (D-Ala-D-Ala) is replaced by a new insensitive one (D-Ala-D-Lac), thus reducing the affinity of vancomycin to its target by three orders of magnitude [61]. The emergence of vancomycin-resistant pathogens (especially VRSA, vancomycin-resistant *S. aureus*) has prompted research for second-generation glycopeptides [55,57]. Promising molecules include semi-synthetic lipoglycopeptide derivatives of natural GPAs such as telavancin, dalbavancin, and oritavancin and recently discovered natural compounds such as corbomycin and complestatin. Telavancin was derived from vancomycin by chemical modification, the addition of a (decylaminoethyl) hydrophobic tail on the vancosamine amino group, and a hydrophilic moiety [(phosphonomethyl)aminomethyl] group at the 4-position of the aromatic amino acid [55,56,59]. The introduction of a hydrophobic side chain serves as a membrane anchor, which not only leads to increased affinity to the pentapeptide termini of Lipid II but also creates a mechanism whereby the antibiotic dissolves into the membrane and makes it more permeable [56,57]. The hydrophilic substituent group has no direct effect on bacterial cells, but it enhances antibacterial potency and tissue distribution, increases the efficiency of elimination via the urinary system, and thus reduces liver and kidney accumulation [62]. Oritavancin, developed as semi-synthetic N–acyl derivative of the glycopeptide chloroeremomycin, differs from vancomycin in its glycosylation pattern. It has enhanced antimicrobial activity due to a dual-action mechanism. Firstly, by enhancing binding to the membrane-associated Lipid II, especially to the pentaglycyl-bridging segment of the PG (unique to staphylococci), thus maintaining an affinity for the modified Lipid II in vancomycin-resistant strains. Secondly, the lipophilic biphenyl moiety disrupts the integrity of the bacterial membrane, leading to depolarization and increased permeability [55,56,57,59]. Dalbavancin is derived from the natural glycopeptide A40926, a member of the teicoplanin family. Through the amidation of the *C*-terminal carboxyl group with an *N*,*N*-dimethylpropylamine group, the original drug undergoes modification. This enhances its antibacterial activity against staphylococci as it may easily interact with the negative phospholipid groups of the bacterial membrane [56]. Moreover, dalbavancin has a terminally branched dodecyl fatty acid chain linked to the glucosamine moiety via an amide linkage, which helps in membrane anchoring (Figure 3B). It binds to D-Ala-D-Ala, with higher affinity compared to its parent compound due to its ability to form dimers [55,57,59]. Using an in silico approach, based on phylogenic analysis of biosynthetic gene clusters (GBCs), it is possible to identify potential GPAs. However, to avoid the rediscovery of already known compounds or highly similar molecules, additional assumptions are necessary. The search for GBCs that lack known self-resistance genes resulted in the discovery of two novel compounds, corbomycin and complestatin, which are moderately related to vancomycin (Figure 3B). The major breakthrough is a completely different mode of action, although still related to peptidoglycan. In contrast to the above mentioned GPAs, corbomycin and complestatin block the action of autolysins—essential peptidoglycan hydrolases that are required for remodeling of the cell wall during growth. Interestingly, the inhibition results from an interaction between the antibiotic and the peptidoglycan, not between the antibiotic and the enzyme. Targeting the substrate for autolysins ensures low resistance development and makes these compounds an exciting avenue for future development [63].

## 4. Agents Decomposing the Bacterial Cell Wall

The agents described above, except for corbomycin and complestatin, act on the synthesis of PG, thus exhibiting antibacterial activity. Molecules from the second group remodel and disassemble PG. Within this group are proteins encoded in bacteriophages. Virion-associated lysins (VALs) locally “drill a hole” in PG to facilitate the injection of phage genetic material into bacteria, whereas endolysins cause massive degradation of PG, which, together with the lysis of the bacterial cell membrane by holins, results in the complete destruction of bacteria. Analogically, proteinaceous agents of bacterial origin, i.e., autolysins, enable controlled remodeling of PG, indispensable for bacterial cell division, whereas bacteriocins, exemplified by lysostaphins, are produced with the intention to kill other bacteria in the vicinity of bacteriocin’s producers. However, each of the proteins, regardless of their origin and biological role, when used in excess or without control, may have a deleterious effect on bacteria. This opens an avenue to use them as effective antibacterials and an alternative to low-molecular-weight antibiotics, especially to combat drug-resistant pathogens.

### 4.1. Peptidoglycan Targeting Enzymes of Bacteriophage Origin

#### 4.1.1. Endolysins

Endolysins comprise a group of bacteriophage-encoded enzymes produced by large double-stranded DNA viruses to break down the bacterial cell wall peptidoglycan (PG) for progeny virions release at the terminal stage [64,65]. Endolysins are synthesized inside the host–cell, but the vast majority of them lack signal peptide sequences and, therefore, are not actively translocated across the cell membrane to reach their substrate, PG. The release of these canonical endolysins is controlled by a second phage gene product in the phage lytic system, alpha-helix-type channel-forming proteins called holins [66,67]. The proteins are synthesized at a genetically programmed time window in the terminal stage of the phage lytic cycle. Upon reaching critical concentration, the hydrophobic holin monomers spontaneously assemble into oligomers and form membrane lesions or holes, through which endolysins accumulated in the cytoplasm are released. Their action leads to PG degradation, osmolysis, and the release of progeny phage particles [68,69,70,71]. A minor part of endolysins (referred to as exported endolysins or e-endolysins), exemplified by Lys44 encoded by *Oenococcus oeni* phage fOg44 and coliphage PI lysozyme Lyz, carry an N-terminal signal peptide that guides their secretion by engaging host transport machinery, most frequently the general secretion pathway (the Sec system) [67,72].

**Figure 4:**
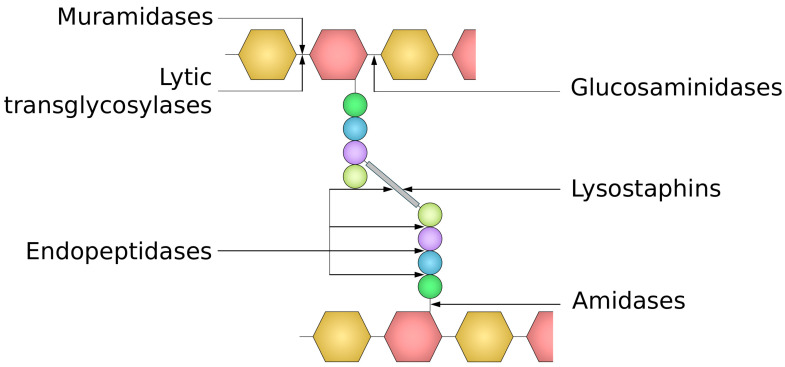
Degradation of peptidoglycan is carried out by endolysins and lysostaphins. The singular monomer of PG contains several glycosidic, amide, and peptide bonds undergoing hydrolysis. Depending on enzymatic activity, endolysins can be divided into five groups. Muramidase (*N*-acetyl-β-d-muramidase) targets the *N*-acetylmuramoyl-β-1,4-*N*-glucosamine bond between MurNAc and GlcNAc (within a single monomer). The same bond is recognized by lytic transglycosylase, but this enzyme’s activity involves non-hydrolytic breakage of the β-1,4-glycosidic bond. Glucosaminidase (*N*-acetyl-glucosaminyl-β-d-glucosaminidase) disrupts glycan strands by cleaving the β-1,4-glysosidic bond between MurNAc and GlcNAc at the reducing end of GlcNAc. Amidase (*N*-acetylmuramoyl-l-alanine) cleaves the peptide bond linking MurNAc with the stem peptide, and endopeptidases also target peptide bonds, but between amino acids of the stem peptide. Interpeptide bridges can be targeted by either endopeptidases or lysostaphins.

The classification of endolysins is based on their target site within the PG structure, with at least nine positions within murein where they are known or are proposed to cleave [73,74]. Depending on their enzymatic specificity, endolysins can be divided into five groups (I−V), targeting glycosidic, amide, or peptide bonds present in PG (Figure 4). Glycosidases (groups I–III) comprise (I) *N*-acetyl-β-d-muramidases (also termed lysozymes), which hydrolyze glycosidic bonds between MurNAc and GlcNAc (N-acetylmuramoyl-β-1,4-*N*-acetylglucosamine bond); (II) *N*-acetyl-glucosaminyl-β-d-glucosaminidases, which cleave linkages between GlcNAc and MurNAc (*N*-acetylglucosaminyl-β-1,4-*N*-acetylmuramine bond at the reducing end of GlcNAc), and thus both catalyze the hydrolysis of β-1-4 glycosidic bond in the glycan strand; and (III) lytic transglycosylases. The latter enzymes are not hydrolases, in contrast to lysozymes and glucosaminidases, since they do not require water to catalyze the reaction. They are very similar to muramidases, as they cleave the same β-1,4 bond between the MurNAc and GlcNAc but involve a nonhydrolytic intramolecular reaction that results in the formation of a 1,6-anhydro ring at the MurNAc residue. Group IV gathers *N*-acetylmuramoyl-l-alanine amidases, performing cleavage of the amide bond between MurNAc and the first highly conserved amino acid residue (L-Ala) of stem peptide. Finally, group (V) contains endopeptidases that cleave peptide bonds between amino acid residues and can be further classified as stem-peptide-specific (e.g., L-alanoyl-d-glutamate endopeptidases, γ-d-glutaminyl-l-lysine endopeptidases), cutting peptide bonds in the stem-peptide-, or interpeptide-bridge-specific endopeptidases (e.g., D-alanyl-glycyl, D-alanyl-L-alanyl endopeptidase), breaking bonds in the cross-bridge [4,74,75,76]. Among these classes, muramidases and amidases seem to be the most commonly found and universal enzymes, cleaving highly conserved bonds in the PG [77]. Typically, endolysins display only one type of enzymatic activity, but there are also bifunctional ones, harboring two independent muralytic activities [64,76]. This double activity (usually endopeptidase and amidase) is a common feature of endolysins (e.g., phi11, phi12, LysK, MV-L, and LysH5) encoded by staphylococcal phages [78,79,80,81].

**Figure 5:**
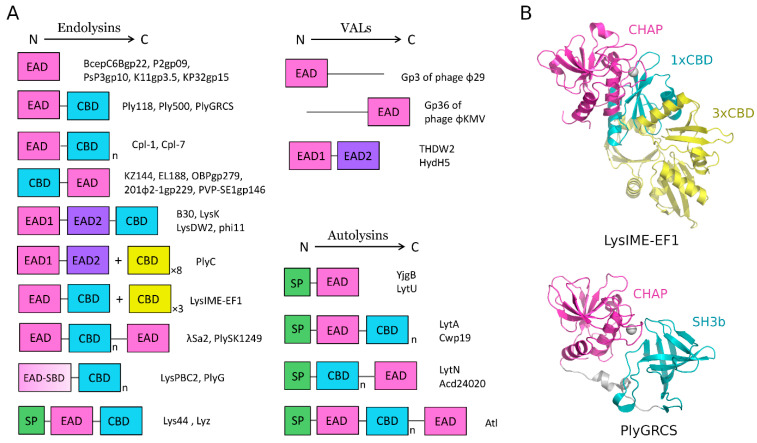
The diversity of different groups of peptidoglycan hydrolases (PGHs). (**A**) Various architectures of PGHs with the names of their representatives. SP, signal peptide; EAD, enzymatically active domain; CBD, cell-wall-binding domain; SBD, spore-binding domain. (**B**) The molecular structure of PlyPSA and LysIME–EF1 endolysins. PDB ID: 8H1I and 6IST, respectively. The coloring corresponds to the first panel. For clarity, three of the four CBDs of LysIME–EF1 are colored yellow. The catalytic Ca^2+^ ions located within CHAP domains are depicted as gray spheres.

Endolysins, to perform cleavage, first have to bind to their substrate. This is achieved by their modular structure, which is a typical feature for the enzymes encoded by phages infecting Gram-positive bacteria [77]. Most of the endolysins studied to date consist of two clearly separated functional domains: (I) the N-terminal enzymatically active catalytic domain (EAD), harboring the active site and catalyzing the breakdown of specific peptidoglycan bonds; and (II) the cell-wall-binding domain (CBD), located at the C-terminal and targeting the enzyme to its specific substrate by recognizing and binding non-covalently to certain ligands in the bacterial cell wall. Both domains are connected to each other by a flexible interdomain linker region of variable length, as exemplified by PlyGRCS endolysin (Figure 5A,B) [82,83,84].

Enzymatically active domains (EADs) have a number of structural motifs, which reflect their activity. The most common include glycosidases (GH24, GH25, GH108), globular muramidase (MURA), phage–lysozyme (LYSO), soluble lytic transglycosylase (SLT), transglycosylase (TRANG), glucosaminidase (GLUCO), amidases (AMI-2, AMI-3, AMI-5, AMI02-C), and peptidases such as cysteine/histidine-dependent amidohydrolase/peptidase (CHAP), NLPC/P60, and PET–M23 motifs. In the case of CBDs, various conserved binding motifs targeting murein or other components of the cell wall have also been found. They include PG-1 and PG-3 type motifs, LYSM domain, SH3 domains with different subtypes, various three-helical bundles (Cpl-7), choline-binding modules (Cpl-1), and others [85,86]. Except for endolysins with a classic domain arrangement, exemplified by enzymes encoded by phages infecting bacteria from *Streptococcus*, *Listeria*, *Clostridioides*, and *Bacillus* genera, [87,88] there are many non-canonical ones. These exhibit more complex architectures, featuring multiple EADs and/or CBDs in different positions (Figure 5A). The combination of two *N*-terminal EADs (endopeptidase followed by amidase) and one C-terminal CBD (SH3) is quite frequent for endolysins from staphylococcal phages origin [89]. Bifunctional streptococcal endolysin B30 has endopeptidase (CHAP) and muramidase catalytic domains, followed by the SH3b domain [90]. However, it has been shown for several dual-EAD endolysins that one of these catalytic domains is virtually inactive or silent, at least in vitro, whereas the other one is dominant and exhibits high lytic activity. For example, the amidase domain of the anti-staphylococcal endolysins phi11 and LysK and the muramidase domain of streptococcal lysin B30 have poor activity and slightly contribute to lysis, but their CHAP domains are highly active and even alone are sufficient to kill the target bacteria [78,90,91,92]. The *Streptococcus agalactiae* prophage endolysin λSa2 comprises two centrally located CBDs (2x Cpl-7) and two flanking EADs (*N*-terminal AMI-5 and *C*-terminal AMI-4) [93]. PlySK1249 endolysin has a similar architecture, which harbors a central CBD surrounded by an *N*-terminal amidase and a *C*-terminal CHAP domain [94]. Endolysins LysPBC2 and PlyG encoded by phages infecting spore-forming bacteria (*Bacillus cereus* and *B. anthracis*, respectively) have an extra spore-binding domain (SBD) within the frame of the catalytic domain (EAD), which specifically binds to spores but not vegetative cells [95]. *S. pneumoniae* phage endolysins, Cpl-1 and Cpl-7, have one EAD with *N*-acetylmuramidase activity and multiple CBDs with six and three choline-binding repeats, respectively [96,97]. LysIME–EF1 from *Enterococcus faecalis* phage exhibits a unique architecture in which one full-length LysIME–EF1 forms a tetramer with its three additional *C*-terminal CBDs synthesized as separate proteins from the alternative start codon within a single gene transcript (Figure 5B) [98]. In contrast, PlyC endolysin, encoded by antistreptococcal phages, is a unique multimeric protein consisting of two separate gene products. The holoenzyme is composed of PlyA, with two EADs exhibiting CHAP and glycoside hydrolase activity and eight PlyB cell-wall-binding subunits (PDB ID: 4F88) [99,100]. The described modular architecture is typical for endolysins from Gram-positive-bacteria-specific phages, whereas endolysins from Gram-negative-bacteria-specific phages are mostly small globular proteins with a single catalytic domain [101,102]. This reflects the differences in cell wall architecture between these major bacterial groups (Figure 5A). Endolysins from phages infecting Gram-negative bacteria (e.g., BcepC6gp22 from *Burkholderia cepacia* phage BcepC6B, P2gp09 from *Escherichia coli* phage P2, PsP3gp10 from *Salmonella enterica* phage PsP3, and K11gp3.5 and KP32gp15 from *Klebsiella pneumoniae* phages K11 and KP32, respectively) are released to the periplasmic space, and their further diffusion is restricted by the outer membrane [103]. But there are also exceptions, like KZ144, EL188, OBPgp279, and 201φ2-1gp229 endolysins, which are encoded by phages infecting bacteria from *Pseudomonas* genus or PVP–SE1gp146 (from *S. enterica* phage PVP–SE1) and exhibit a modular structure with an inverse orientation of domains, with an *N*-terminal CBD and a *C*-terminal EAD [104].

Efficient cell wall cleavage by endolysins involves CBD-dependent recognition and binding to specific carbohydrate ligands of conserved modules in the bacterial cell wall, which positions the catalytic domain for the particular substrate. This ensures a certain degree of specificity of the target enzyme, since the binding substrates occur only in endolysin-sensitive bacteria, thus significantly influencing the range of activity of the entire enzyme. CBD targets include intact PG or its subunits, or secondary cell-wall polymers like *N*-acetylglucosamine, choline, polyrhamnose, (lipo)teichoic acids, neutral polysaccharides, and proteins [85,105,106]. The presence of a specific target for CBDs within the cell wall is often restricted to particular bacterial species or even strains, imposing a level of specificity and thus activity of endolysins [107]. CBDs attach to these ligands noncovalently with high specificity and affinity, which is comparable to the affinity of antibodies [108]. This high binding-specific recognition property was elegantly demonstrated on CBDs of *Listeria monocytogenes* phage endolysins, Ply118 and Ply500, and CBD from antistaphylococcal LysP108 fused with green fluorescent protein [108,109]. Also, likely because of the high affinity of the enzymes to their substrates in the cell wall, antibodies against phage endolysins obtained from immunized animals have little or no neutralizing effect on the antibacterial activity of endolysin, as shown in many studies, both in vitro and in vivo [80,110,111]. Nevertheless, there are also numerous reports on *C*-terminally truncated lysin constructs where the *N*-terminal lytic domain maintains its activity, or is even higher than in full-length endolysin, showing that CBD is not always essential for its antibacterial activity. This was demonstrated for the anti-staphylococcal endolysins LysK and phi11 [91,112,113]. The loss in CBDs may decrease access of the truncated endolysins to the cell wall targets and result in limited activity, but on the other hand, it could also lead to the broadening of the lytic spectra against non-host organisms, such as related species or serovars, by alleviating the limitations of CBD specificity [114]. For example, full-length endolysin LysPBC1 from a phage infecting only some *B. cereus* strains showed stronger lytic activity against the host cells than the truncated LysPBC1; however, the EAD showed higher lytic activity than the full-length LysPBC1 against nonnative targets, such as *B. subtilis*, *B. pumilus*, and *B. licheniformis* [115]. The activity and efficiency of the catalytic domains, regardless of the presence of CBD, can be explained by their net positive charge and affinity for the generally negatively charged surface of Gram-positive cells. Negatively charged EADs without CBDs may not efficiently bind and hydrolyze negatively charged PG. Even with functional CBDs, lysins with negatively charged EADs may be less active than those that carry net positive charges [114,116,117]. Thus, endolysin activity and specificity are not conferred solely by CBD, as EAD may also contribute to them, provided that there is a molecular target for EAD in the cell wall.

#### 4.1.2. Virion-Associated Lysins

Besides endolysins, viral cell-wall-targeting agents also include exogenous *trans*-acting virion-associated lysins (VALs), which could be considered as a possible source of next-generation antibacterial agents as well. These peptidoglycan-degrading enzymes, compared to endolysins, have not been as extensively studied and systematically analyzed regarding their biochemical properties, PG-degrading activity, and therapeutic application as potential antibacterials [118]. VALs are also known as virion-associated peptidoglycan hydrolases (VAPGHs), tail-associated muralytic enzymes (TAME), tail-associated lysins (TAL), or exolysins. These enzymes are encoded by some double-stranded DNA phages infecting both Gram-negative and Gram-positive bacteria. In contrast to endolysins, they participate in local peptidoglycan degradation during phage particle adsorption on the bacterial surface, which triggers major virion conformational changes that place VALs in close contact with the bacterial cell wall, allowing the phage to inject its DNA into the host–cell [119].

The vast majority of VALs do not have recognizable CBDs. The presence of this domain is, however, dispensable because the contact between VAL and cell wall PG substrate is guaranteed by the interaction of other phage proteins and host–cell surface receptors [67]. Although VALs act on PG ‘from without’ and endolysins ‘from within’, EADs of VALs share a high degree of amino acid similarity to endolysins’ EADs, suggesting a similar mode of action in cleavage mechanisms and hydrolyzing specific and highly conserved bonds in murein [120,121]. The majority of VALs have a single lytic domain fused to the phage capsid structural protein, which can be located at the *N*-terminus (Gp3 of phage φ29) or *C*-terminus (Gp36 of phage φKMV) (Figure 5A) [119]. The most common EADs in VALs exhibit glycosidase and endopeptidase activities [67]. However, in phages infecting Gram-positive bacteria, there are also VALs carrying two catalytic domains with distinct cleavage specificities [119,122]. For example, *S. aureus* phage DW2 has a VAL THDW2, with an *N*-terminus CHAP domain followed by a muramidase. Interestingly, the phage also encodes bifunctional LysDW2 endolysin with CHAP and amidase activity [123].

### 4.2. Peptidoglycan Targeting Enzymes of Bacterial Origin

#### 4.2.1. Autolysins

Autolysins comprise a group of bacterial-origin cis-acting enzymes targeting PG. Bacterial cells utilize autolysins for different physiological functions, which include cell wall remodeling during cell growth, separation and division, biofilm formation, toxin release, sporulation, germination, peptidoglycan recycling, and programmed death (autolysis). Therefore, these enzymes are not only redundant but also tightly controlled in bacteria [118,124]. Autolysins have a modular architecture and contain a catalytic domain (EAD) and a cell-wall-binding domain (CBD). However, in contrast to endolysins, they also contain a signal peptide (SP) sequence at the *N*-terminus for their export outside the cell or to the periplasm (Figure 5A) [4,120]. Since autolysins locally disassemble PG, their EADs have to possess an activity reversing events during PG synthesis. These include glucosaminidases, muramidases, lytic transglycosylases, amidases, and peptidases with NlpC/P60, CHAP, or PET–M23 motifs. Among CBDs, the most frequent are LysM, choline-binding domain (ChBD), SH3b, and GW modules [125]. An autolysin, Acd24020, from *C. difficile*, possesses a *C*-terminal endopeptidase catalytic domain belonging to the NlpC/P60 family and three SH3b CBDs at the *N*-terminus. However, the truncated form of Acd24020 with only EAD also has full lytic activity (PDB ID: 7CFL) [124]. *S. aureus* autolysins LytU (PDB ID: 5KQB) [126] and LytM (PDB ID: 1QWY) [127] also have EAD with endopeptidase activity. Autolysins with transglycosylase and N-acetylglucosaminidase EADs are present in Cwp19 and AtlA from *C. difficile* and *E. faecalis*, respectively [128,129]. In contrast, the major autolysin (Atl) of *S. epidermidis* (AtlE) and *S. aureus* (AtlS) is bifunctional, with *N*-terminal amidase and *C*-terminal glucosaminidase domains and repeat domains, R1a, R1b, R2a, R2b, R3a, and R3b, located between EADs. Some of these domains have been shown to bind to lipoteichoic acids, thus performing the role of CDBs. The full-length Atl undergoes proteolytic processing to generate two independent extracellular peptidoglycan hydrolases involved in the partitioning of daughter cells after cell division [130]. There are also non-modular enzymes, having EAD only, as exemplified by *Lactococcus lactis* autolysin YjgB with the NlpC/P60 domain [131].

Although autolysins degrade PG, they are essential for the functioning of bacteria. As such, they may be a target for antibacterial drugs that inhibit their activity. On the other hand, if not tightly controlled or used exogenously, autolysins might also be considered highly specific antimicrobials acting by massively lysing PG, reducing bacterial virulence, or modulating the response of the host immune system. In *S. aureus*, disruption of autolysin LytN results in cell wall structural alterations, affecting cellular morphology and growth defects. In contrast, its overexpression causes cell lysis and death [132]. Pneumococcal LytA autolysin was successfully applied to treat mice with β-lactam-resistant pneumococcal peritonitis–sepsis [133,134]. Certain autolysins have been pointed out as virulence factors in Gram-positive bacteria. In the case of *S. aureus*, the major autolysin Atl has been associated with bacterial cell adhesion to different host ligands. Atl-mediated PG hydrolysis, in particular during daughter cell separation, was shown to be critical for maintaining optimal levels of *S. aureus* surface cell-wall-anchored proteins (e.g., fibronectin-binding proteins (FnBPs) and IgG-binding protein A (Spa)) important for virulence. It was shown that disrupting Atl function with the glycopeptide antibiotic complestatin, which binds to peptidoglycan and blocks autolysins’ activity, or by using a catalytically inactive Atl mutant reduced the level of surface cell-wall-anchored proteins and consequently decreased the affinity of *S. aureus* for host–cell ligands. This negatively impacted the early stages of bacterial colonization in a systemic model of *S. aureus* infection [135]. Moreover, Atl was shown to trim the exposed ends of the peptidoglycan molecules on the surface of the cell wall, which could otherwise be detected by the host. In the fruit fly *Drosophila*-infection model, the *S. aureus Δatl* mutant had a decreased ability to kill flies due to its binding by peptidoglycan-recognition proteins and induction of the host immune response. However, the mutant bacteria were able to evade the immune system after they had been treated (“shaved”) with the purified autolysin [136]. Barriers to therapeutic applications of endogenous autolysins are their tightly regulated, lower intrinsic activity, and suboptimal targeting of the bacterial cell wall compared to endolysins [137]. However, these limitations can be overcome by creating modified molecules by domain shuffling or fusion with other CBDs. Enhancing the antibacterial potential of chimeric endogenous autolysins was demonstrated on *S. aureus* LytM, a zinc-dependent glycyl–glycine endopeptidase lacking CBD, which was fused to the CBD from lysostaphin LSs (see below). While native LytM exogenously added to *S. aureus* is only marginally bactericidal, its fusion with the lysostaphin CBD enhances its anti-staphylococcal activity over 500 times [138]. Recently, a bioinformatics pipeline called LEDGOs (lytic enzyme domains grouped by organism) has been developed. The algorithm was applied to systematically find, analyze, and characterize autolysin-building blocks and their architectures within and across a set of different important pathogenic bacteria. Moreover, the analysis indicates sequences that would likely benefit from experimental characterization, highlighting potential targets for chemotherapeutic intervention and suggesting strategies for creating next-generation autolysins [139].

#### 4.2.2. Lysostaphins—*Staphylococcus*-Genus-Specific Bacteriocins

**Figure 6:**
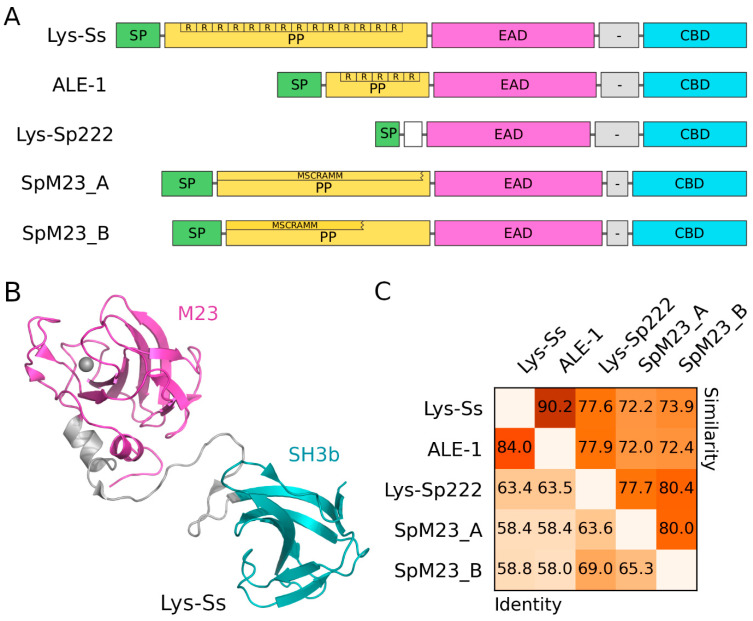
Structure and similarity of staphylococcal lysostaphins. (**A**) The most common lysostaphin architecture includes an N-terminal signal peptide (SP) followed by a propeptide (PP), which contains short 13-amino-acid-long repeats (R) or a putative MSCRAMM domain. The essential part of each lysostaphin comprises an enzymatically active domain and a cell-wall-binding domain, separated by a short linker (EAD–CBD). (**B**) The molecular structure of the mature form of Lys–Ss includes the EAD and CBD domains (M23-SH3b), with a Zn^2+^ ion located within the M23 metalloproteinase domain, depicted as a gray sphere. The coloring corresponds to the first panel. PDB ID: 4LXC. (**C**) Sequence identity and similarity of the EAD–CBD regions among known staphylococcal lysostaphins. Lys–Ss and ALE-1 sequences are highly similar, while sequence similarity among other lysostaphins is moderate. SP, signal peptide; PP, propeptide; R, repeat; EAD, enzymatically active domain; CBD, cell-wall-binding domain.

A number of strains belonging to the *Staphylococcus* genus eliminate other competing staphylococci by secreting peptidoglycan hydrolases called lysostaphins into their environment. Those enzymes are specifically glycylglycine (Gly–Gly) endopeptidases that target the pentaglycine bridge of the staphylococcal peptidoglycan, consequently leading to cell wall lysis. In terms of protein architecture, lysostaphins always possess an N-terminal signal peptide (SP), which directs the protein for secretion outside the cell. In most known lysostaphins, the SP is followed by a propeptide (PP) that is removed in the extracellular environment to render a mature and active enzyme [140,141]. The last two domains, which are the only ones present in the mature enzyme form, are enzymatically active and cell-wall-binding domains separated by a short linker (EAD–CBD) [140,141,142,143]. The EAD is a zinc metallopeptidase M23 domain (PF01551) responsible for peptidoglycan hydrolysis, while the *C*-terminally located CBD is an SH3b domain (PF08460), which binds to the cell wall [144,145,146,147,148]. The prevalence of lysostaphin producers among staphylococci is not high and has, to date, been reported for only a limited number of strains belonging to five staphylococcal species. The longest-known lysostaphin (Lys–Ss) was originally described by Schindler and Schuhardt [149]. It was reported to be a factor secreted by a Gram-positive coccus, distinctive from lysozyme and bacteriolytic towards *S. aureus*. The producer was soon after identified as *S. simulans* [150], and Lys–Ss was found to be encoded in the pACK1 plasmid [151]. Lys–Ss protein architecture is the most typical for lysostaphins (Figure 6A,B), consisting of an SP followed by a relatively long PP and the EAD–CBD region [140,143]. Notably, Lys–Ss PP contains 14 repeats of a 13-amino-acid-long motif. The activities of Lys–Ss EAD and CBD domains have been studied in detail. It was demonstrated that EAD targets the pentaglycine bridge [152,153] and CBD binds to the entire peptide part (the peptide stem and the pentaglycine bridge) of the staphylococcal peptidoglycan [154,155,156,157]. Over three decades after the discovery of Lys–Ss, no other distinctive lysostaphin had been reported. Then, the plasmid-encoded ALE-1, originating from *S. capitis*, was purified and reported to be a glycylglycine endopeptidase [142]. ALE-1 has a similar architecture to Lys–Ss; however, its PP is much shorter and contains only 5 repeats of a 13-amino-acid-long motif, the sequence of which is different from that of Lys–Ss (Figure 6A). Markedly, ALE-1 PP does not undergo any processing [142]. Nevertheless, the EAD–CBD regions of Lys–Ss and ALE-1 display quite a high similarity (Figure 6C). Recently, our group characterized a lysostaphin, Lys–Sp222, which originates from *S. pseudintermedius*. Notably, Lys–Sp222 displays moderate activity against human skin commensal *S. epidermidis* while being highly active against the notorious human pathogen *S. aureus* [158]. In contrast to previously described lysostaphins, Lys–Sp222 is encoded in the bacterial chromosome. Architectonically, Lys–Sp222 is similar to Lys–Ss and ALE-1; however, it does not contain any PP (Figure 6A). The similarity of the EAD–CBD regions of Lys–Sp222 to the corresponding regions of Lys–Ss and ALE-1 is moderate (Figure 6C). The year we discovered Lys–Sp222, a study on SpM23_A and SpM23_B was released. Both lysostaphins are chromosome-encoded and occur in *S. pettenkoferi* and the closely related *S. argensis* [141]. Interestingly, SpM23_A and SpM23_B are reported to contain an MSCRAMM family domain located between the SP and the EAD–CBD region. However, our search of the InterPro database does not indicate any domain to be present in this region, and a search of NCBI Conserved Domain Database (CDD) reports such domains as non-specific and partial hits. Moreover, the authors demonstrate this region to have inhibitory activity on SpM23_A and SpM23_B, which suggests it is simply a PP. However, the PPs of these two lysostaphins do not contain any repeats, making them clearly different from the corresponding regions found in Lys–Ss and ALE-1. Nevertheless, the architecture of SpM23_A and SpM23_B corresponds well to the general architecture of lysostaphins. The similarity of the EAD–CBD regions between both lysostaphins, as well as the similarity of this region to the corresponding ones in other lysostaphins, is moderate (Figure 6C). Lysostaphin producers utilize a very straightforward immunity mechanism against their own weapon. Since the pentaglycine bridge is the target, immunity is achieved by altering this structure. There are three major staphylococcal peptidyl transferases involved in the synthesis of the pentaglycine bridge. All of them use glycyl–tRNA as a substrate to sequentially add glycine residues to the growing bridge [159]. FmhB, also known as FemX, adds the first glycine residue to the peptide stem [160]. The second and third residues are added by FemA, while the fourth and fifth residues are added by FemB [161,162,163]. The mechanism of lysostaphin immunity has been particularly well-studied for Lys–Ss. In the vicinity of the Lys–Ss gene, a gene coding for a lysostaphin immunity factor (*lif*), also known as the endopeptidase resistance gene (*epr*), is located. Lif is an enzyme that complements FemB activity, meaning that in the presence of Lif, the activity of FemB is redundant. Similarly to FemB, Lif utilizes aminoacyl–tRNA to introduce subsequent amino acid residues to the pentaglycine bridge. However, Lif utilizes seryl–tRNA and introduces serine instead of glycine residues. This simple alternation renders the cell wall of a Lys–Ss producer immune to the enzyme [143,164,165,166,167]. Similar immunity factors have been reported to accompany lysostaphins other than Lys–Ss [141,142,158,167,168]. Taking all five known lysostaphins into consideration, it is worth mentioning that most of the research on lysostaphins has been primarily undertaken on Lys–Ss. More recently characterized lysostaphins, such as Lys–Sp222 and SpM23_A and B, have not yet been directly demonstrated to target the pentaglycine bridge, although their activity is assumed based on homology. Moreover, Lys–Ss is the only lysostaphin whose complete molecular structure has been determined so far [147].

## 5. Enzymes Targeting Cell Wall as Potential Antibacterials: Spectrum of Activity and Advantages over Antibiotics

### 5.1. Resistance to Peptidoglycan Hydrolases

Most bacteria can develop resistance mechanisms to protect themselves against the action of antibacterial agents. These mechanisms tend to inactivate the agents by degradation, decreasing their intracellular concentration either by reduced membrane permeability or active efflux pumps. Moreover, bacteria may modify targets of antimicrobial agents, making them insensitive to their action [169]. At the moment, based on numerous studies, developing bacterial resistance to the activity of the endolysins is unlikely to readily emerge de novo, giving the hope that these enzymes could be a long-term solution in the treatment of antibiotic-resistant pathogens. The attempts to generate and identify resistant bacterial strains were based on experiments where cells were repeatedly exposed to low concentrations of native or engineered endolysins, and were performed on different bacterial cells and enzymes [65,83]. For instance, *Streptococcus pneumoniae* were repeatedly subjected to low concentrations of the Pal endolysin either on agar plates or in liquid culture, but no strains with resistant phenotypes emerged even after numerous cycles [170]. Similar experiments were conducted with PlyG endolysin and *Bacillus anthracis* [171]. Also, attempts to create methicillin-resistant *S. aureus* (MRSA) resistant to the chimeric endolysin ClyS were unsuccessful. Bacteria exposed to this enzyme did not develop any resistance, nor did ClyS lose potency with repeat administration [172]. Though sub-lethal exposure of *S. aureus* to LysK and lysostaphin increased their minimal inhibitory concentration (MIC) 42 and 585-fold, respectively. However, treatment of the bacteria with different variants of chimeric proteins formed by the fusion of two catalytic domains from LysK; one of lysostaphin showed hardly any increase in resistance [173] and neither did the successive exposure of *S. aureus* to subinhibitory concentrations of the chimeric Ply187 endolysin [174]. Even the random mutagenesis of endolysin-sensitive strains could not promote the development of resistant cells, while the approach easily induced bacterial resistance to classical antibiotics [121]. This suggests that the coevolution of phages and their hosts over the millennia has led to the selection of endolysins binding to and cleaving unique, highly conserved, and immutable targets in the cell wall, which are essential for cell viability. This allows phages to avoid becoming trapped inside, and to guarantee escape from the host cells, presumably making the formation of endolysin-resistance quite unlikely or more difficult compared to conventional antibiotics [75,116]. Moreover, the extracellular nature of the target PG also limits the number of possible mechanisms of resistance to endolysins. However, single examples of bacteria losing sensitivity to endolysins applied exogenously (‘lysis from without’) have been described [175].

Despite these encouraging results with endolysins, it should be noted that similar exposures to other antibacterials targeting PG resulted in the generation of mutants resistant to vancomycin, lysozyme, and lysostaphin [176,177,178]. The resistance mechanism is based on chemical and structural modifications of PG, which could include *N*-deacetylation of GlcNAc and/or MurNAc sugars, *O*-acetylation or *N*-glycolylation of MurNAc, and *D*-alanylation/*O*-acetylation of teichoic acids. Moreover, alterations in envelope charge and integrity and in crosslinking of the peptide stem is responsible for resistance to lysozyme. Modifications within the pentaglycine cross-bridge of several *S. aureus* strains drives resistance to lysostaphin [165,176].

However, the likelihood of the development of antimicrobial resistance could be theoretically decreased by using enzymes with two catalytic domains with different peptidoglycan specificity. This would require two simultaneous compensatory mutations in the same cell and is believed to be a rare event. This also gives room for designing and engineering new chimeric proteins, to improve the properties of natural lysins, increasing their activity, modifying specificity spectrum, and improving physico-chemical properties [179]. Furthermore, the use of different antimicrobials, preferably acting synergistically, increases the effectiveness against the target bacteria and allows for a reduction in the required dose of each antimicrobial compound [180,181].

### 5.2. Lysins as Potential Antibacterials

By digesting PG, endolysins cause immediate lysis of bacteria, especially in regard to Gram-positive cells. When applied exogenously, as recombinant proteins, to bacterial cells, they become potent, alternative antibacterials, frequently called enzybiotics [179,182,183]. Endolysins are generally active against the bacterial genera associated with their phages, i.e., an endolysin originating from a streptococcal phage will specifically target streptococci, and endolysin from a staphylococcal phage will act against staphylococci, etc. Such specificity and high lytic effectiveness open up the possibility of their application against many well-known and fatal Gram-positive infectious bacteria, as reported for *Streptococcus* spp. (*S. gordonii*, *S. equi*, *S. mutans*, *S. sanguinis*, *S. pyogenes*, *S. agalactiae*, *S. dysgalactiae*, *S. pneumoniae*, *S. infantis*, *S. anginosus*, *S. suis*, *S. oralis*, *S. mitis*, *S. uberis*) [88,111,184]; *Staphylococcus* spp. (*S. aureus*, *S. epidermidis*) [89,185]; *Enterococcus* spp. (*E. faecalis*, *E. faecium*) [186,187]; *Clostridioides* spp. (*C. difficile*, *C. perfringens*, *C. tyrobutyricum. C. sporogenes*) [188,189,190]; *Bacillus* spp. (*B. anthracis*, *B. cereus*) [171]; *Mycobacterium* spp. [191]; and *Listeria monocytogenes* [192]. Endolysins inhibiting the growth of Gram-negative pathogens, including *Pseudomonas aeruginosa*, *Acinetobacter baumannii*, *Escherichia coli*, *Salmonella* Typhimurium, *Stenotrophomonas maltophilia*, *Burkholderia* spp., *Klebsiella pneumoniae* and *Helicobacter pylori*, are also known [193,194].

However, there are also endolysins with a broader spectrum of lytic activity. The canonical example is the enterococcal endolysin PlyV12 (from *Enterococcus faecalis* phage φ1), which, in addition to the host strain, is capable of lysing clinical and laboratory *E. faecalis* and *E. faecium* strains, streptococci (*S. pyogenes*, group B and C streptococci) and staphylococci (*S. aureus*) [186]. LysAB2 endolysin from the *Acinetobacter baumannii* phage also has a wide spectrum of antibacterial activity, including not only the host but also *S. aureus*, *B. subtilis*, *S. sanguis*, *E. coli*, *Citrobacter freundii*, and *Salmonella enterica* [195]. Similarly, endolysin Mur-LH exhibits lytic action mainly against thermophilic lactobacilli but also lactococci, pediococci, *B. subtilis*, *Brevibacterium* spp., and *E. faecium* [196]. λSa1 and λSa2 enzymes lysed the cell walls of *S. agalactiae*, *S. pneumoniae*, and *S. aureus* [93]. LysPBC2 showed very broad lytic activity against bacteria from *Bacillus*, *Listeria*, and *Clostridioides* genera [95]. It is worth noting that endolysins’ lytic activity also extends to antibiotic-resistant bacterial strains [80,110,186,197,198].

This potential use of lysins as antibacterial therapeutic agents has been demonstrated successfully in a variety of studies, ranging from the killing of drug-resistant bacteria in simple antibacterial assays to the effective elimination of bacterial biofilms in vitro and in vivo, ending with the eradication of pathogens in animal models of bacterial infection. PG-targeting enzymes can be applied alone or in synergistic mode with other lysins having different enzymatic specificities or antibacterial agents such as bacteriocins and classical antibiotics [169,199,200].

Analogically to antibiotics, generations are also distinguished among lysins. First-generation ones are natural enzymes, some of which are currently ongoing in the first clinical trials. The second-generation lysins are enzymes with improved antibacterial and biochemical properties, such as expanded activities towards Gram-negative pathogens and higher stability. In the third-generation lysins, protein-engineering efforts focus on improving properties relevant for in vivo application, such as pharmacokinetics and/or pharmacodynamics, in addition to improvements in bioavailability, antibacterial activity, half-life, and reducing pro-inflammatory responses [201]. Improving physico-chemical and antimicrobial properties can be achieved by mutagenesis, and domain shuffling, thus creating chimeric proteins (chimerolysins and artificial lysins) with novel and desired properties [179]. In the post-antibiotic era where a number of multi-drug-resistant strains increase, described peptidoglycan hydrolases, both wild-type and their engineered variants, serve as a source of potential novel antimicrobial agents.

The specificity of most endolysins and lysostaphins to particular species or genera is a key advantage over conventional antibiotics, which tend to have broad antimicrobial effects across multiple species. Therefore, PG-targeting lysins, as a novel class of antibacterial agents, display a number of highly desirable properties and advantages over antibiotics. First, they offer a new mode of antimicrobial action as “enzybiotics” with high target selectivity, low effective molar dosages, extremely high efficiency, and rapid killing kinetics. Second, the lysins exhibit activity against bacteria regardless of their antibiotic sensitivity, including multidrug-resistant pathogens, and synergy with other antibacterial agents (including conventional antibiotics). Furthermore, they display narrow-spectrum antibacterial activity without affecting the commensal microbiota and potentially beneficial micro-organisms. At the same time, the lysins retain the ability to kill pathogens that colonize mucosal surfaces, tissues, and blood, as well as those that form biofilms, regardless of the physiological state of these bacteria. In addition, they demonstrate relative safety, no/low toxicity and immunogenicity, and efficacy without apparent side effects. Importantly, there is a low likelihood of bacteria developing resistance to PG-targeting lysins. Finally, their modular structure and potential for domain shuffling through genetic engineering allow for their further optimization and refinement.

### 5.3. Lysostaphins as Potential Antibacterials

The idea of Lys–Ss as an antibacterial agent to complement or replace classic antibiotics in the therapy of staphylococcal infections emerged soon after its discovery. An important advantage of Lys–Ss is the efficacy against non-diving cells, which are usually insensitive to antibiotics, as well as against encapsulated ones [202,203]. Since the discovery of Lys–Ss, dozens of studies demonstrating its efficacy in eradicating pathogenic *S. aureus* in animal models have been published [204,205]. The first in vivo studies were performed on mice infected either intraperitoneally or intravenously and administered with Lys–Ss intravenously. These studies indicated high anti-staphylococcal activity of Lys–Ss in vivo and showed efficacy exceeding that of antibiotics, even when the antibiotics were administered multiple times following the infection. Moreover, no adverse immunological effects from repeated intravenous administration of Lys–Ss were observed in mice or rabbits [206,207,208]. An investigation conducted decades later on the in vivo activity of Lys–Ss in rabbit models of keratitis and aortic valve endocarditis caused by methicillin-resistant *S. aureus* (MRSA) supported these findings. In the latter study, Lys–Ss proved to be more effective than vancomycin; however, the combination of both was found to be the most effective. Lys–Ss also demonstrated greater efficacy than vancomycin in clearing MRSA infections in a neonatal mouse model. Similar to the initial studies that used animal infection models, no adverse immunological reactions caused by Lys–Ss administration were observed, even when subjects were immunized against Lys–Ss prior to the experiments [209,210,211]. The development of anti-staphylococcal drugs based on Lys–Ss may be accelerated by recent advances in recombinant lysostaphin production, engineering a non-immunogenic Lys–Ss with T cell epitopes removed, creating conjugates of Lys–Ss and PEG that are characterized by significantly improved serum half-life, and manufacturing Lys–Ss-coated materials [212,213,214,215,216]. Notably, *S. aureus* is not only a clinically relevant human pathogen but also a species that causes significant economic losses in the food industry, particularly as a causative agent of mastitis in dairy cattle. It has been proposed that transgenic animals producing Lys–Ss might be a solution to limit staphylococcal infections in livestock. For instance, transgenic mice secreting engineered non-glycosylated Lys–Ss into milk have been demonstrated to be substantially resistant to intramammary staphylococcal infections [217]. Undoubtedly, utilizing Lys–Ss as an anti-staphylococcal drug will be of high importance not only in human healthcare but also in global food production. Nevertheless, regarding clinical applications of Lys–Ss, the number of studies in animal models greatly exceeds the number of actual clinical trials in humans. There are two studies from the time of Lys–Ss discovery demonstrating its efficacy when administered topically to nasal carriers [218,219]. Only one case study describes a systemic administration of Lys–Ss in a neutropenic patient suffering from staphylococcal pneumonia with metastatic abscesses, who died three days after receiving Lys–Ss. The death was not related to the staphylococcal infection, and samples collected ante- and post-mortem demonstrated eradication of MRSA infection [220]. Regarding more recent research, information on only one clinical trial focused on Lys–Ss application in humans can be found on the WHO International Clinical Trials Registry Platform (CTRI/2016/09/007277, last refreshed in 2021). This Phase IIb/III randomized study, funded by Bharat Biotech International Ltd. in India, aims to evaluate the efficacy and safety of a topical recombinant lysostaphin gel formulation in subjects with uncomplicated *S. aureus* (including MRSA) skin and skin structure infections. Given the rapidly growing multidrug resistance among bacteria [1], it is expected that the number of clinical trials focused on lysostaphins will increase significantly in the near future. Lysostaphins other than Lys–Ss are still awaiting investigation of their activity in animal models and humans. Lys–Sp222 is particularly interesting in this context due to its high activity against *S. aureus*, a notorious pathogen of both humans and livestock, and its much lower activity towards the commensal *S. epidermidis* [158].

## 6. Conclusions

The antibiotics and proteins mentioned above have different origins and natural functions. Virion-associated lysins (VALs) are responsible for phages entering their bacterial hosts, while endolysins enable them to escape. Antibiotics and bacteriocins participate in “microbial warfare” against each other, and autolysins are involved in physiological cell wall remodeling. They all target the peptidoglycan (PG), which is essential for the proper functioning of bacteria. Classical low-molecular-weight antibiotics, although still widely used, are ineffective against a growing number of multi-drug-resistant bacteria. A gap in the discovery of novel antibiotics has led to a focus on proteinaceous agents produced by bacteria themselves or encoded by bacteriophages with potential antibacterial activity. PG, as the outermost layer of Gram-positive bacteria, is an attractive target for novel antibacterials. Unlike antibiotics, which work by suppressing the synthesis of PG, peptidoglycan hydrolases applied externally attack the polymer directly, leading to its degradation. Because the bonds targeted by these enzymes are often crucial for maintaining the PG structure and are highly conserved, it is unlikely that bacteria will develop resistance to their action.

Peptidoglycan-targeting enzymes have a modular organization with a wide variety of enzymatically active domains (EADs) and cell-wall-binding domains (CBDs) acting as “building blocks” with different catalytic and binding properties. This, combined with the extensive number and variety of these enzymes from natural sources, provides almost unlimited opportunities for searching and engineering these proteins as potential new biotherapeutics. These proteins could have broad activity or species-specificity, offering a way to combat clinically relevant multi-drug-resistant bacteria from *Staphylococcus*, *Streptococcus*, and *Enterococcus* genera.

## Data Availability

No new data were created in this study. Data sharing is not applicable to this article.

## Abbreviations

AMI—amidase domain; CBD—cell-wall-binding domain; CDD—Conserved Domain Database; CHAP—cysteine/histidine-dependent amidohydrolase/peptidase domain; EAD—enzymatically active domain; ESBLs—extended-spectrum β-lactamases; FnBPs—fibronectin-binding proteins; GBCs—biosynthetic gene clusters; GH—glycosidase domain; GlcNAc—N-acetylglucosamine; GLUCO—glucosaminidase domain; GPAs—glycopeptide antibiotics; LEDGOs—lytic enzyme domains grouped by organism; LYSO—phage-lysozyme domain; Lys–Sp222—lysostaphin from *S. pseudintermedius*; Lys–Ss—lysostaphin from *S. simulans*; MBLs—metallo-β-lactamases; m-DAP—meso-diaminopimelate; MDR—multidrug-resistant; MIC—minimal inhibitory concentration; MRSA—methicillin-resistant *S. aureus*; MSSA—methicillin-sensitive *S. aureus*; MURA—muramidase domain; MurNAc—N-acetylmuramic acid; NLPC/P60—new lipoprotein C from *E. coli* and protein of 60 kDa of *Listeria monocytogenes* domain; PBPs—penicillin-binding proteins; PDB—Protein Data Bank; PEG—polyethylene glycol; PET-M23—peptidase domain M23; PG—peptidoglycan; PGHs—peptidoglycan hydrolases; PP—propeptide; SBD—spore-binding domain; SBLs—serine-β-lactamases; SCCmec—staphylococcal cassette chromosome mec; SH3b—Src homology 3 bacterial domain; SLT—soluble lytic transglycosylase domain; SP—signal peptide; TAL—tail-associated lysins; TAME—tail-associated muralytic enzymes; TG—transglycosylase; TP—transpeptidase; TRANG—transglycosylase domain; UDP—uridine diphosphate; Und-P—undecaprenyl phosphate lipid carrier; VALs—virion-associated lysins; VAPGHs—virion-associated peptidoglycan hydrolases; VRSA—vancomycin-resistant *S. aureus*; WHO—World Health Organization.

## References

[1] WHO (2024). WHO Bacterial Priority Pathogens List, 2024.

[2] Silhavy T.J., Kahne D., Walker S. (2010). The bacterial cell envelope. Cold Spring Harb. Perspect. Biol..

[3] Garde S., Chodisetti P.K., Reddy M. (2021). Peptidoglycan: Structure, Synthesis, and Regulation. EcoSal Plus.

[4] Vollmer W., Joris B., Charlier P., Foster S. (2008). Bacterial peptidoglycan (murein) hydrolases. FEMS Microbiol. Rev..

[5] Egan A.J.F., Errington J., Vollmer W. (2020). Regulation of peptidoglycan synthesis and remodelling. Nat. Rev. Microbiol..

[6] Galinier A., Delan-Forino C., Foulquier E., Lakhal H., Pompeo F. (2023). Recent Advances in Peptidoglycan Synthesis and Regulation in Bacteria. Biomolecules.

[7] Rajagopal M., Walker S. (2017). Envelope structures of Gram-positive bacteria. Current Topics in Microbiology and Immunology.

[8] Rajguru V., Chatterjee S., Garde S., Reddy M. (2024). Crosslink cleaving enzymes: The smart autolysins that remodel the bacterial cell wall. Trends Microbiol..

[9] Lobanovska M., Pilla G. (2017). Penicillin’s Discovery and Antibiotic Resistance: Lessons for the Future?. Yale J. Biol. Med..

[10] Duerden B.I. (2012). MRSA: Why have we got it and can we do anything about it?. Eye.

[11] Bunnell K., Duong A., Ringsred T., Mian A., Bhathena S. (2022). Aminopenicillins for treatment of ampicillin-resistant enterococcal urinary tract infections. Am. J. Health Pharm..

[12] Florin T.A., Byczkowski T., Gerber J.S., Ruddy R., Kuppermann N. (2020). Diagnostic testing and antibiotic use in young children with community-acquired pneumonia in the United States, 2008–2015. J. Pediatr. Infect. Dis. Soc..

[13] Hussen N.H., Hamid S.J., Sabir M.N., Hasan A.H., Mohammed S.J., Shali A.A.K. (2024). Novel Penicillin Derivatives Against Selected Multiple-drug Resistant Bacterial Strains: Design, Synthesis, Structural Analysis, In Silico and In Vitro Studies. Curr. Org. Synth..

[14] Chaudhry S.B., Veve M.P., Wagner J.L. (2019). Cephalosporins: A Focus on Side Chains and β-Lactam Cross-Reactivity. Pharmacy.

[15] Lin X., Kück U. (2022). Cephalosporins as key lead generation beta-lactam antibiotics. Appl. Microbiol. Biotechnol..

[16] Duplessis C., Crum-Cianflone N.F. (2011). Ceftaroline: A New Cephalosporin with Activity Against Methicillin-Resistant *Staphylococcus aureus* (MRSA). Clin. Med. Rev. Ther..

[17] Quiñonez-Flores A., Martinez-Guerra B.A., Román-Montes C.M., Tamez-Torres K.M., González-Lara M.F., Ponce-de-León A., Rajme-López S. (2024). Cephalotin Versus Dicloxacillin for the Treatment of Methicillin-Susceptible *Staphylococcus aureus* Bacteraemia: A Retrospective Cohort Study. Antibiotics.

[18] Werth B.J., Sakoulas G., Rose W.E., Pogliano J., Tewhey R., Rybak M.J. (2013). Ceftaroline increases membrane binding and enhances the activity of daptomycin against daptomycin-nonsusceptible vancomycin-intermediate *Staphylococcus aureus* in a pharmacokinetic/pharmacodynamic model. Antimicrob. Agents Chemother..

[19] Jiao F., Bao Y., Li M., Zhang Y., Zhang F., Wang P., Tao J., Tong H.H.Y., Guo J. (2023). Unraveling the mechanism of ceftaroline-induced allosteric regulation in penicillin-binding protein 2a: Insights for novel antibiotic development against methicillin-resistant Staphylococcus aureus. Antimicrob. Agents Chemother..

[20] Noel G.J. (2007). Clinical profile of ceftobiprole, a novel β-lactam antibiotic. Clin. Microbiol. Infect..

[21] Méndez R., Latorre A., González-Jiménez P. (2022). Ceftobiprole medocaril. Rev. Esp. Quimioter..

[22] Wi Y.M., Kwon K.T., Jeon C.H., Kim S.H., Hwang S., Bae S., Kim Y., Chang H.H., Kim S.W., Cheong H.S. (2023). Carbapenem Use in the Last Days of Life: A Nationwide Korean Study. Antibiotics.

[23] Queenan A.M., Bush K. (2007). Carbapenemases: The versatile β-lactamases. Clin. Microbiol. Rev..

[24] Armstrong T., Fenn S.J., Hardie K.R. (2021). JMM Profile: Carbapenems: A broad-spectrum antibiotic. J. Med. Microbiol..

[25] Mansour H., El Ouweini A., Chahine E.B., Karaoui L.R. (2021). Imipenem/cilastatin/relebactam: A new carbapenem β-lactamase inhibitor combination. Am. J. Health Pharm..

[26] O’Donnell J.N., Lodise T.P. (2022). New Perspectives on Antimicrobial Agents: Imipenem-Relebactam. Antimicrob. Agents Chemother..

[27] Bassetti M., Magnè F., Giacobbe D.R., Bini L., Vena A. (2022). New antibiotics for Gram-negative pneumonia. Eur. Respir. Rev..

[28] Thu Z.M., Sun J., Ji J., He L., Ji J., Iqbal Z., Myo K.K., Gao Y., Zhai L., Mu Y. (2021). Synthesis and antibacterial evaluation of new monobactams. Bioorg. Med. Chem. Lett..

[29] Chen W., Zhang Y.M., Davies C. (2017). Penicillin-binding protein 3 is essential for growth of *Pseudomonas aeruginosa*. Antimicrob. Agents Chemother..

[30] Freischem S., Grimm I., López-Pérez A., Willbold D., Klenke B., Vuong C., Dingley A.J., Weiergräber O.H. (2021). Interaction mode of the novel monobactam AIC499 targeting penicillin binding protein 3 of Gram-negative bacteria. Biomolecules.

[31] Blais J., Lopez S., Li C., Ruzin A., Ranjitkar S., Dean C.R., Leeds J.A., Casarez A., Simmons R.L., Reck F. (2018). In vitro activity of LYS228, a novel monobactam antibiotic, against multidrug-resistant enterobacteriaceae. Antimicrob. Agents Chemother..

[32] Sun Y., Liao X., Huang Z., Xie Y., Liu Y., Ma C., Jiang B., Zhang L., Yan Y., Li G. (2020). Therapeutic effect and mechanisms of the novel monosulfactam 0073. Antimicrob. Agents Chemother..

[33] Li Z., Guo Z., Lu X., Ma X., Wang X., Zhang R., Hu X., Wang Y., Pang J., Fan T. (2023). Evolution and development of potent monobactam sulfonate candidate IMBZ18g as a dual inhibitor against MDR Gram-negative bacteria producing ESBLs. Acta Pharm. Sin. B.

[34] Schalk I.J. (2018). A Trojan-Horse Strategy Including a Bacterial Suicide Action for the Efficient Use of a Specific Gram-Positive Antibiotic on Gram-Negative Bacteria. J. Med. Chem..

[35] Meini M.R., Llarrull L.I., Vila A.J. (2014). Evolution of metallo-β-lactamases: Trends revealed by natural diversity and in vitro evolution. Antibiotics.

[36] Bush K., Jacoby G.A. (2010). Updated functional classification of β-lactamases. Antimicrob. Agents Chemother..

[37] Philippon A., Slama P., Dény P., Labia R. (2016). A structure-based classification of class A β-Lactamases, a broadly diverse family of enzymes. Clin. Microbiol. Rev..

[38] Eiamphungporn W., Schaduangrat N., Malik A.A., Nantasenamat C. (2018). Tackling the antibiotic resistance caused by class a β-lactamases through the use of β-lactamase inhibitory protein. Int. J. Mol. Sci..

[39] Yagi T., Wachino J.I., Kurokawa H., Suzuki S., Yamane K., Doi Y., Shibata N., Kato H., Shibayama K., Arakawa Y. (2005). Practical methods using boronic acid compounds for identification of class C β-lactamase-producing *Klebsiella pneumoniae* and *Escherichia coli*. J. Clin. Microbiol..

[40] Philippon A., Arlet G., Labia R., Iorga B.I. (2022). Class C β-Lactamases: Molecular Characteristics. Clin. Microbiol. Rev..

[41] Kaitany K.C.J., Klinger N.V., June C.M., Ramey M.E., Bonomo R.A., Powers R.A., Leonard D.A. (2013). Structures of the class D carbapenemases OXA-23 and OXA-146: Mechanistic basis of activity against carbapenems, extended-spectrum cephalosporins, and aztreonam. Antimicrob. Agents Chemother..

[42] Antunes N.T., Lamoureaux T.L., Toth M., Stewart N.K., Frase H., Vakulenko S.B. (2014). Class D β-lactamases: Are they all carbapenemases?. Antimicrob. Agents Chemother..

[43] Kim Y., Maltseva N., Wilamowski M., Tesar C., Endres M., Joachimiak A. (2020). Structural and biochemical analysis of the metallo-β-lactamase L1 from emerging pathogen *Stenotrophomonas maltophilia* revealed the subtle but distinct di-metal scaffold for catalytic activity. Protein Sci..

[44] Boyd S.E., Livermore D.M., Hooper D.C., Hope W.W. (2020). Metallo-β-lactamases: Structure, function, epidemiology, treatment options, and the development pipeline. Antimicrob. Agents Chemother..

[45] López-Agudelo V.A., Gómez-Ríos D., Ramirez-Malule H. (2021). Clavulanic acid production by streptomyces clavuligerus: Insights from systems biology, strain engineering, and downstream processing. Antibiotics.

[46] Mauri C., Maraolo A.E., Di Bella S., Luzzaro F., Principe L. (2021). The revival of aztreonam in combination with avibactam against metallo-β-lactamase-producing Gram-negatives: A systematic review of in vitro studies and clinical cases. Antibiotics.

[47] Lahiri S.D., Johnstone M.R., Ross P.L., McLaughlin R.E., Olivier N.B., Alm R.A. (2014). Avibactam and class C β -lactamases: Mechanism of inhibition, conservation of the binding pocket, and implications for resistance. Antimicrob. Agents Chemother..

[48] Sauvage E., Kerff F., Terrak M., Ayala J.A., Charlier P. (2008). The penicillin-binding proteins: Structure and role in peptidoglycan biosynthesis. FEMS Microbiol. Rev..

[49] Fishovitz J., Hermoso J.A., Chang M., Mobashery S. (2014). Penicillin-binding protein 2a of methicillin-resistant *Staphylococcus aureus*. IUBMB Life.

[50] Otero L.H., Rojas-Altuve A., Llarrull L.I., Carrasco-López C., Kumarasiri M., Lastochkin E., Fishovitz J., Dawley M., Hesek D., Lee M. (2013). How allosteric control of *Staphylococcus aureus* penicillin binding protein 2a enables methicillin resistance and physiological function. Proc. Natl. Acad. Sci. USA.

[51] Hunashal Y., Kumar G.S., Choy M.S., D’Andréa É.D., Da Silva Santiago A., Schoenle M.V., Desbonnet C., Arthur M., Rice L.B., Page R. (2023). Molecular basis of β-lactam antibiotic resistance of ESKAPE bacterium *E. faecium* Penicillin Binding Protein PBP5. Nat. Commun..

[52] Bartsch A., Ives C.M., Kattner C., Pein F., Diehn M., Tanabe M., Munk A., Zachariae U., Steinem C., Llabrés S. (2021). An antibiotic-resistance conferring mutation in a neisserial porin: Structure, ion flux, and ampicillin binding. Biochim. Biophys. Acta—Biomembr..

[53] Abavisani M., Kodori M., Akrami F., Radfar A., Hashemi A. (2022). Relationships between Efflux Pumps and Emergence of Heteroresistance: A Comprehensive Study on the Current Findings. Can. J. Infect. Dis. Med. Microbiol..

[54] Wu W., Huang J., Xu Z. (2024). Antibiotic influx and efflux in *Pseudomonas aeruginosa*: Regulation and therapeutic implications. Microb. Biotechnol..

[55] Butler M.S., Hansford K.A., Blaskovich M.A.T., Halai R., Cooper M.A. (2014). Glycopeptide antibiotics: Back to the future. J. Antibiot..

[56] Sarkar P., Yarlagadda V., Ghosh C., Haldar J. (2017). A review on cell wall synthesis inhibitors with an emphasis on glycopeptide antibiotics. Medchemcomm.

[57] Binda E., Marinelli F., Marcone G.L. (2014). Old and new glycopeptide antibiotics: Action and resistance. Antibiotics.

[58] Nicolaou K.C., Rigol S. (2018). A brief history of antibiotics and select advances in their synthesis. J. Antibiot..

[59] Blaskovich M.A.T., Hansford K.A., Butler M.S., Jia Z., Mark A.E., Cooper M.A. (2018). Developments in Glycopeptide Antibiotics. ACS Infect. Dis..

[60] Wu W., Liu M., Geng J., Wang M. (2021). Teicoplanin combined with conventional vancomycin therapy for the treatment of pulmonary methicillin-resistant *Staphylococcus aureus* and *Staphylococcus epidermidis* infections. World J. Clin. Cases.

[61] Jovetic S., Zhu Y., Marcone G.L., Marinelli F., Tramper J. (2010). β-Lactam and glycopeptide antibiotics: First and last line of defense?. Trends Biotechnol..

[62] Leadbetter M.R., Adams S.M., Bazzini B., Fatheree P.R., Karr D.E., Krause K.M., Lam B.M.T., Linsell M.S., Nodwell M.B., Pace J.L. (2004). Hydrophobic vancomycin derivatives with improved ADME properties: Discovery of telavancin (TD-6424). J. Antibiot..

[63] Culp E.J., Waglechner N., Wang W., Fiebig-Comyn A.A., Hsu Y.P., Koteva K., Sychantha D., Coombes B.K., Van Nieuwenhze M.S., Brun Y.V. (2020). Evolution-guided discovery of antibiotics that inhibit peptidoglycan remodelling. Nature.

[64] Fischetti V.A. (2005). Bacteriophage lytic enzymes: Novel anti-infectives. Trends Microbiol..

[65] Schmelcher M., Donovan D.M., Loessner M.J. (2012). Bacteriophage endolysins as novel antimicrobials. Future Microbiol..

[66] Fernández-García L., Blasco L., Lopez M., Bou G., García-Contreras R., Wood T., Tomas M. (2016). Toxin-antitoxin systems in clinical pathogens. Toxins.

[67] São-José C. (2018). Engineering of phage-derived lytic enzymes: Improving their potential as antimicrobials. Antibiotics.

[68] Wang I.N., Deaton J., Young R. (2003). Sizing the holin lesion with an endolysin-β-galactosidase fusion. J. Bacteriol..

[69] Wang I.N., Smith D.L., Young R. (2000). Holins: The protein clocks of bacteriophage infections. Annu. Rev. Microbiol..

[70] Vukov N., Moll I., Bläsi U., Scherer S., Loessner M.J. (2003). Functional regulation of the *Listeria monocytogenes* bacteriophage A118 holin by an intragenic inhibitor lacking the first transmembrane domain. Mol. Microbiol..

[71] Young R. (2013). Phage lysis: Do we have the hole story yet?. Curr. Opin. Microbiol..

[72] Borysowski J., Weber-Dąbrowska B., Górski A. (2006). Bacteriophage endolysins as a novel class of antibacterial agents. Exp. Biol. Med..

[73] Payne K.M., Hatfull G.F. (2012). Mycobacteriophage endolysins: Diverse and modular enzymes with multiple catalytic activities. PLoS ONE.

[74] Hermoso J.A., García J.L., García P. (2007). Taking aim on bacterial pathogens: From phage therapy to enzybiotics. Curr. Opin. Microbiol..

[75] Gutiérrez D., Fernández L., Rodríguez A., García P. (2018). Are phage lytic proteins the secret weapon to kill *Staphylococcus aureus*?. MBio.

[76] Love M.J., Abeysekera G.S., Muscroft-Taylor A.C., Billington C., Dobson R.C.J. (2020). On the catalytic mechanism of bacteriophage endolysins: Opportunities for engineering. Biochim. Biophys. Acta—Proteins Proteom..

[77] Loessner M.J. (2005). Bacteriophage endolysins—Current state of research and applications. Curr. Opin. Microbiol..

[78] Sass P., Bierbaum G. (2007). Lytic activity of recombinant bacteriophage φ11 and φ12 endolysins on whole cells and biofilms of *Staphylococcus aureus*. Appl. Environ. Microbiol..

[79] O’Flaherty S., Coffey A., Meaney W., Fitzgerald G.F., Ross R.P. (2005). The recombinant phage lysin LysK has a broad spectrum of lytic activity against clinically relevant staphylococci, including methicillin-resistant *Staphylococcus aureus*. J. Bacteriol..

[80] Rashel M., Uchiyama J., Ujihara T., Uehara Y., Kuramoto S., Sugihara S., Yagyu K.I., Muraoka A., Sugai M., Hiramatsu K. (2007). Efficient elimination of multidrug-resistant *Staphylococcus aureus* by cloned lysin derived from bacteriophage φMR11. J. Infect. Dis..

[81] Obeso J.M., Martínez B., Rodríguez A., García P. (2008). Lytic activity of the recombinant staphylococcal bacteriophage ΦH5 endolysin active against *Staphylococcus aureus* in milk. Int. J. Food Microbiol..

[82] Schmelcher M., Loessner M.J. (2016). Bacteriophage endolysins: Applications for food safety. Curr. Opin. Biotechnol..

[83] Love M.J., Bhandari D., Dobson R.C.J., Billington C. (2018). Potential for bacteriophage endolysins to supplement or replace antibiotics in food production and clinical care. Antibiotics.

[84] Krishnappa G., Mandal M., Ganesan S., Babu S., Padavattan S., Haradara Bahubali V.K., Padmanabhan B. (2023). Structural and biochemical insights into the bacteriophage PlyGRCS endolysin targeting methicillin-resistant *Staphylococcus aureus* (MRSA) and serendipitous discovery of its interaction with a cold shock protein C (CspC). Protein Sci..

[85] Oliveira H., Melo L.D.R., Santos S.B., Nóbrega F.L., Ferreira E.C., Cerca N., Azeredo J., Kluskens L.D. (2013). Molecular Aspects and Comparative Genomics of Bacteriophage Endolysins. J. Virol..

[86] Broendum S.S., Buckle A.M., McGowan S. (2018). Catalytic diversity and cell wall binding repeats in the phage-encoded endolysins. Mol. Microbiol..

[87] Korndörfer I.P., Danzer J., Schmelcher M., Zimmer M., Skerra A., Loessner M.J. (2006). The Crystal Structure of the Bacteriophage PSA Endolysin Reveals a Unique Fold Responsible for Specific Recognition of Listeria Cell Walls. J. Mol. Biol..

[88] Wong K.Y., Megat Mazhar Khair M.H., Song A.A.L., Masarudin M.J., Chong C.M., In L.L.A., Teo M.Y.M. (2022). Endolysins against Streptococci as an antibiotic alternative. Front. Microbiol..

[89] Haddad Kashani H., Schmelcher M., Sabzalipoor H., Seyed Hosseini E., Moniri R. (2018). Recombinant endolysins as potential therapeutics against antibiotic-resistant *Staphylococcus aureus*: Current status of research and novel delivery strategies. Clin. Microbiol. Rev..

[90] Donovan D.M., Foster-Frey J., Dong S., Rousseau G.M., Moineau S., Pritchard D.G. (2006). The cell lysis activity of the *Streptococcus agalactiae* bacteriophage B30 endolysin relies on the cysteine, histidine-dependent amidohydrolase/peptidase domain. Appl. Environ. Microbiol..

[91] Donovan D.M., Lardeo M., Foster-Frey J. (2006). Lysis of staphylococcal mastitis pathogens by bacteriophage phi11 endolysin. FEMS Microbiol. Lett..

[92] Becker S.C., Dong S., Baker J.R., Foster-Frey J., Pritchard D.G., Donovan D.M. (2009). LysK CHAP endopeptidase domain is required for lysis of live staphylococcal cells. FEMS Microbiol. Lett..

[93] Pritchard D.G., Dong S., Kirk M.C., Cartee R.T., Baker J.R. (2007). LambdaSa1 and LambdaSa2 prophage lysins of *Streptococcus agalactiae*. Appl. Environ. Microbiol..

[94] Oechslin F., Daraspe J., Giddey M., Moreillon P., Resch G. (2013). In vitro characterization of PlySK1249, a novel phage lysin, and assessment of its antibacterial activity in a mouse model of *Streptococcus agalactiae* bacteremia. Antimicrob. Agents Chemother..

[95] Kong M., Na H., Ha N.C., Ryu S. (2018). LysPBC2, a novel endolysin harboring a *Bacillus cereus* spore binding domain. Appl. Environ. Microbiol..

[96] Alreja A.B., Linden S.B., Lee H.R., Chao K.L., Herzberg O., Nelson D.C. (2023). Understanding the Molecular Basis for Homodimer Formation of the Pneumococcal Endolysin Cpl-1. ACS Infect. Dis..

[97] Silva-Martin N., Molina R., Angulo I., Mancheño J.M., García P., Hermoso J.A. (2010). Crystallization and preliminary crystallographic analysis of the catalytic module of endolysin from Cp-7, a phage infecting *Streptococcus pneumoniae*. Acta Crystallogr. Sect. F Struct. Biol. Cryst. Commun..

[98] Zhou B., Zhen X., Zhou H., Zhao F., Fan C., Perčulija V., Tong Y., Mi Z., Ouyang S. (2020). Structural and functional insights into a novel two-component endolysin encoded by a single gene in *Enterococcus faecalis* phage. PLoS Pathog..

[99] Nelson D., Schuch R., Chahales P., Zhu S., Fischetti V.A. (2006). PlyC: A multimeric bacteriophage lysin. Proc. Natl. Acad. Sci. USA.

[100] McGowan S., Buckle A.M., Mitchell M.S., Hoopes J.T., Gallagher D.T., Heselpoth R.D., Shen Y., Reboul C.F., Law R.H.P., Fischetti V.A. (2012). X-ray crystal structure of the streptococcal specific phage lysin PlyC. Proc. Natl. Acad. Sci. USA.

[101] Wladyka B., Bonar E., Savini V. (2018). Application of Staphylococci in the Food Industry and Biotechnology.

[102] Schmelcher M., Powell A.M., Becker S.C., Camp M.J., Donovan D.M. (2012). Chimeric phage lysins act synergistically with lysostaphin to kill mastitis-causing *Staphylococcus aureus* in murine mammary glands. Appl. Environ. Microbiol..

[103] Walmagh M., Boczkowska B., Grymonprez B., Briers Y., Drulis-Kawa Z., Lavigne R. (2013). Characterization of five novel endolysins from Gram-negative infecting bacteriophages. Appl. Microbiol. Biotechnol..

[104] Drulis-Kawa Z., Majkowska-Skrobek G., Maciejewska B., Delattre A.-S., Lavigne R. (2013). Learning from Bacteriophages—Advantages and Limitations of Phage and Phage-Encoded Protein Applications. Curr. Protein Pept. Sci..

[105] Ganguly J., Low L.Y., Kamal N., Saile E., Forsberg L.S., Gutierrez-Sanchez G., Hoffmaster A.R., Liddington R., Quinn C.P., Carlson R.W. (2013). The secondary cell wall polysaccharide of *Bacillus anthracis* provides the specific binding ligand for the C-terminal cell wall-binding domain of two phage endolysins, PlyL and PlyG. Glycobiology.

[106] Fischetti V.A. (2003). Novel method to control pathogenic bacteria on human mucous membranes. Ann. N. Y. Acad. Sci..

[107] Pastagia M., Schuch R., Fischetti V.A., Huang D.B. (2013). Lysins: The arrival of pathogen-directed anti-infectives. J. Med. Microbiol..

[108] Loessner M.J., Kramer K., Ebel F., Scherer S. (2002). C-terminal domains of *Listeria monocytogenes* bacteriophage murein hydrolases determine specific recognition and high-affinity binding to bacterial cell wall carbohydrates. Mol. Microbiol..

[109] Yang H., Xue J., Li J., Hu G., Li H., Lu S., Fu Z. (2022). Green fluorescent protein-fused bacteriophage cellular wall-binding domain as broad-spectrum signal probe for fluorimetry of methicillin-resistant *Staphylococcus aureus* strains. Anal. Chim. Acta.

[110] Jado I., López R., García E., Fenoll A., Casal J., García P., Pallares R., de la Campa A.G., Bouza E., Baquero F. (2003). Phage lytic enzymes as therapy for antibiotic-resistant *Streptococcus pneumoniae* infection in a murine sepsis model. J. Antimicrob. Chemother..

[111] Linden S.B., Alreja A.B., Nelson D.C. (2021). Application of bacteriophage-derived endolysins to combat streptococcal disease: Current State and perspectives. Curr. Opin. Biotechnol..

[112] Horgan M., O’Flynn G., Garry J., Cooney J., Coffey A., Fitzgerald G.F., Paul Ross R., McAuliffe O. (2009). Phage lysin LysK can be truncated to its CHAP domain and retain lytic activity against live antibiotic-resistant staphylococci. Appl. Environ. Microbiol..

[113] Alaksandr Ž., Sergey G., Maksim P., Sergey K., Niyaz S., Uladzimir P., Mikhail S. (2020). Efficient matrix-assisted refolding of the recombinant anti-staphylococcal truncated endolysin LysKCA and its structural and enzymatic description. Protein Expr. Purif..

[114] Van Tassell M.L., Angela Daum M., Kim J.S., Miller M.J. (2016). Creative lysins: *Listeria* and the engineering of antimicrobial enzymes. Curr. Opin. Biotechnol..

[115] Kong M., Ryu S. (2015). Bacteriophage PBC1 and its endolysin as an antimicrobial agent against *Bacillus cereus*. Appl. Environ. Microbiol..

[116] Donovan D.M. (2007). Bacteriophage and Peptidoglycan Degrading Enzymes with Antimicrobial Applications. Recent Pat. Biotechnol..

[117] Low L.Y., Yang C., Perego M., Osterman A., Liddington R. (2011). Role of net charge on catalytic domain and influence of cell wall binding domain on bactericidal activity, specificity, and host range of phage lysins. J. Biol. Chem..

[118] Bhagwat A., Mixon M., Collins C.H., Dordick J.S. (2020). Opportunities for broadening the application of cell wall lytic enzymes. Appl. Microbiol. Biotechnol..

[119] Latka A., Maciejewska B., Majkowska-Skrobek G., Briers Y., Drulis-Kawa Z. (2017). Bacteriophage-encoded virion-associated enzymes to overcome the carbohydrate barriers during the infection process. Appl. Microbiol. Biotechnol..

[120] Wu X., Kwon S.J., Kim J., Kane R.S., Dordick J.S. (2017). Biocatalytic nanocomposites for combating bacterial pathogens. Annu. Rev. Chem. Biomol. Eng..

[121] Roach D.R., Donovan D.M. (2015). Antimicrobial bacteriophage-derived proteins and therapeutic applications. Bacteriophage.

[122] Rodríguez-Rubio L., Martínez B., Donovan D.M., Rodríguez A., García P. (2013). Bacteriophage virion-associated peptidoglycan hydrolases: Potential new enzybiotics. Crit. Rev. Microbiol..

[123] Keary R., McAuliffe O., Ross R.P., Hill C., O’Mahony J., Coffey A. (2014). Genome analysis of the staphylococcal temperate phage DW2 and functional studies on the endolysin and tail hydrolase. Bacteriophage.

[124] Sekiya H., Tamai E., Kawasaki J., Murakami K., Kamitori S. (2021). Structural and biochemical characterizations of the novel autolysin Acd24020 from *Clostridioides difficile* and its full-function catalytic domain as a lytic enzyme. Mol. Microbiol..

[125] Chapot-Chartier M.P., Konig H., Claus H., Varma A. (2010). Chapter 13 Bacterial Autolysins. Prokaryotic Cell Wall Compounds, Structure and Biochemistry.

[126] Raulinaitis V., Tossavainen H., Aitio O., Juuti J.T., Hiramatsu K., Kontinen V., Permi P. (2017). Identification and structural characterization of LytU, a unique peptidoglycan endopeptidase from the lysostaphin family. Sci. Rep..

[127] Odintsov S.G., Sabala I., Marcyjaniak M., Bochtler M. (2004). Latent LytM at 1.3 Å resolution. J. Mol. Biol..

[128] Wydau-Dematteis S., El Meouche I., Courtin P., Hamiot A., Lai-Kuen R., Saubaméa B., Fenaille F., Butel M.J., Pons J.L., Dupuy B. (2018). Cwp19 is a novel lytic transglycosylase involved in stationary-phase autolysis resulting in toxin release in *Clostridium difficile*. MBio.

[129] Eckert C., Lecerf M., Dubost L., Arthur M., Mesnage S. (2006). Functional analysis of AtlA, the major N-acetylglucosaminidase of *Enterococcus faecalis*. J. Bacteriol..

[130] Götz F., Heilmann C., Stehle T. (2014). Functional and structural analysis of the major amidase (Atl) in *Staphylococcus*. Int. J. Med. Microbiol..

[131] Redko Y., Courtin P., Mézange C., Huard C., Chapot-Chartier M.P. (2007). *Lactococcus lactis* gene YjgB encodes a γ-D-glutaminyl-L-lysyl- endopeptidase which hydrolyzes peptidoglycan. Appl. Environ. Microbiol..

[132] Frankel M.B., Hendrickx A.P.A., Missiakas D.M., Schneewind O. (2011). LytN, a murein hydrolase in the cross-wall compartment of *Staphylococcus aureus*, is involved in proper bacterial growth and envelope assembly. J. Biol. Chem..

[133] Rodríguez-Cerrato V., García P., Huelves L., García E., Del Prado G., Gracia M., Ponte C., López R., Soriano F. (2007). Pneumococcal LytA autolysin, a potent therapeutic agent in experimental peritonitis-sepsis caused by highly β-lactam-resistant *Streptococcus pneumoniae*. Antimicrob. Agents Chemother..

[134] Bonnet J., Durmort C., Jacq M., Mortier-Barrière I., Campo N., VanNieuwenhze M.S., Brun Y.V., Arthaud C., Gallet B., Moriscot C. (2017). Peptidoglycan O-acetylation is functionally related to cell wall biosynthesis and cell division in *Streptococcus pneumoniae*. Mol. Microbiol..

[135] Leonard A.C., Goncheva M.I., Gilbert S.E., Shareefdeen H., Petrie L.E., Thompson L.K., Khursigara C.M., Heinrichs D.E., Cox G. (2023). Autolysin-mediated peptidoglycan hydrolysis is required for the surface display of *Staphylococcus aureus* cell wall-anchored proteins. Proc. Natl. Acad. Sci. USA.

[136] Atilano M.L., Pereira P.M., Vaz F., Catalão M.J., Reed P., Grilo I.R., Sobral R.G., Ligoxygakis P., Pinho M.G., Filipe S.R. (2014). Bacterial autolysins trim cell surface peptidoglycan to prevent detection by the drosophila innate immune system. eLife.

[137] Osipovitch D.C., Therrien S., Griswold K.E. (2015). Discovery of novel *S. aureus* autolysins and molecular engineering to enhance bacteriolytic activity. Appl. Microbiol. Biotechnol..

[138] Osipovitch D.C., Griswold K.E. (2015). Fusion with a cell wall binding domain renders autolysin LytM a potent anti-*Staphylococcus aureus* agent. FEMS Microbiol. Lett..

[139] Mitchell S.J., Verma D., Griswold K.E., Bailey-Kellogg C. (2021). Building blocks and blueprints for bacterial autolysins. PLoS Comput. Biol..

[140] Recsei P.A., Gruss A.D., Novick R.P. (1987). Cloning, sequence, and expression of the lysostaphin gene from *Staphylococcus simulans*. Proc. Natl. Acad. Sci. USA.

[141] Wysocka A., Jagielska E., Łężniak Ł., Sabała I. (2021). Two New M23 Peptidoglycan Hydrolases With Distinct Net Charge. Front. Microbiol..

[142] Sugai M., Fujiwara T., Akiyama T., Ohara M., Komatsuzawa H., Inoue S., Suginaka H. (1997). Purification and molecular characterization of glycylglycine endopeptidase produced by *Staphylococcus capitis* EPK1. J. Bacteriol..

[143] Thumm G., Götz F. (1997). Studies on prolysostaphin processing and characterization of the lysostaphin immunity factor (Lif) of *Stphylococcus simulans* biovar *staphylolyticus*. Mol. Microbiol..

[144] Fujiwara T., Aoki S., Komatsuzawa H., Nishida T., Ohara M., Suginaka H., Sugai M. (2005). Mutation analysis of the histidine residues in the glycylglycine endopeptidase ALE-1. J. Bacteriol..

[145] Rawlings N.D., Morton F.R., Kok C.Y., Kong J., Barrett A.J. (2008). MEROPS: The peptidase database. Nucleic Acids Res..

[146] Hirakawa H., Akita H., Fujiwara T., Sugai M., Kuhara S. (2009). Structural insight into the binding mode between the targeting domain of ALE-1 (92AA) and pentaglycine of peptidoglycan. Protein Eng. Des. Sel..

[147] Sabala I., Jagielska E., Bardelang P.T., Czapinska H., Dahms S.O., Sharpe J.A., James R., Than M.E., Thomas N.R., Bochtler M. (2014). Crystal structure of the antimicrobial peptidase lysostaphin from *Staphylococcus simulans*. FEBS J..

[148] Lu J.Z., Fujiwara T., Komatsuzawa H., Sugai M., Sakon J. (2006). Cell wall-targeting domain of glycylglycine endopeptidase distinguishes among peptidoglycan cross-bridges. J. Biol. Chem..

[149] Schindler C.A., Schuhardt V.T. (1964). Lysostaphin: A New Bacteriolytic Agent for the *Staphylococcus*. Proc. Natl. Acad. Sci. USA.

[150] Sloan G.L., Robinson J.M., Kloos W.E. (1982). Identification of *Staphylococcus staphylolyticus* NRRL B-2628 as a biovar of Staphylococcus simulans. Int. J. Syst. Bacteriol..

[151] Heath L.S., Heath H.E., Sloan G.L. (1987). Plasmid-encoded lysostaphin endopeptidase gene of *Staphylococcus simulans* biovar *staphylolyticus*. FEMS Microbiol. Lett..

[152] Browder H.P., Zygmunt W.A., Young J.R., Tavormina P.A. (1965). Lysostaphin: Enzymatic mode of action. Biochem. Biophys. Res. Commun..

[153] Iversen O.-J., Grov A. (1973). Studies on Lysostaphin: Separation and Characterization of Three Enzymes. Eur. J. Biochem..

[154] Baba T., Schneewind O. (1996). Target cell specificity of a bacteriocin molecule: A C-terminal signal directs lysostaphin to the cell wall of *Staphylococcus aureus*. EMBO J..

[155] Gründling A., Schneewind O. (2006). Cross-linked peptidoglycan mediates lysostaphin binding to the cell wall envelope of *Staphylococcus aureus*. J. Bacteriol..

[156] Mitkowski P., Jagielska E., Nowak E., Bujnicki J.M., Stefaniak F., Niedziałek D., Bochtler M., Sabała I. (2019). Structural bases of peptidoglycan recognition by lysostaphin SH3b domain. Sci. Rep..

[157] Gonzalez-Delgado L.S., Walters-Morgan H., Salamaga B., Robertson A.J., Hounslow A.M., Jagielska E., Sabała I., Williamson M.P., Lovering A.L., Mesnage S. (2020). Two-site recognition of *Staphylococcus aureus* peptidoglycan by lysostaphin SH3b. Nat. Chem. Biol..

[158] Bonar E., Bukowski M., Chlebicka K., Madry A., Bereznicka A., Kosecka-Strojek M., Dubin G., Miedzobrodzki J., Mak P., Wladyka B. (2021). Human skin microbiota-friendly lysostaphin. Int. J. Biol. Macromol..

[159] Kamiryo T., Matsuhashi M. (1972). The biosynthesis of the cross-linking peptides in the cell wall peptidoglycan of *Staphylococcus aureus*. J. Biol. Chem..

[160] Rohrer S., Ehlert K., Tschierske M., Labischinski H., Berger-Bachi B. (1999). The essential *Staphylococcus aureus* gene *fmhB* is involved in the first step of peptidoglycan pentaglycine interpeptide formation. Proc. Natl. Acad. Sci. USA.

[161] Kopp U., Roos M., Wecke J., Labischinski H. (1996). Staphylococcal peptidoglycan interpeptide bridge biosynthesis: A novel antistaphylococcal target?. Microb. Drug Resist..

[162] Ehlert K., Schroder W., Labischinski H. (1997). Specificities of FemA and FemB for different glycine residues: FemB cannot substitute for FemA in staphylococcal peptidoglycan pentaglycine side chain formation. J. Bacteriol..

[163] Strandén A.M., Ehlert K., Labischinski H., Berger-BÄCHI B. (1997). Cell wall monoglycine cross-bridges and methicillin hypersusceptibility in a *femAB* null mutant of methicillin-resistant *Staphylococcus aureus*. J. Bacteriol..

[164] Robinson J.M., Hardman J.K., Sloan G.L. (1979). Relationship between lysostaphin endopeptidase production and cell wall composition in *Staphylococcus staphylolyticus*. J. Bacteriol..

[165] DeHart H.P., Heath H.E., Heath L.S., LeBlanc P.A., Sloan G.L. (1995). The lysostaphin endopeptidase resistance gene (epr) specifies modification of peptidoglycan cross bridges in *Staphylococcus simulans* and *Staphylococcus aureus*. Appl. Environ. Microbiol..

[166] Tschierske M., Ehlert K., Strandén A.M., Berger-Bächi B. (1997). Lif, the lysostaphin immunity factor, complements FemB in staphylococcal peptidoglycan interpeptide bridge formation. FEMS Microbiol. Lett..

[167] Ehlert K., Tschierske M., Mori C., Schröder W., Berger-Bächi B. (2000). Site-specific serine incorporation by Lif and Epr into positions 3 and 5 of the staphylococcal peptidoglycan interpeptide bridge. J. Bacteriol..

[168] Sugai M., Fujiwara T., Ohta K., Komatsuzawa H., Ohara M., Suginaka H. (1997). *epr*, which encodes glycylglycine endopeptidase resistance, is homologous to *femAB* and affects serine content of peptidoglycan cross bridges in *Staphylococcus capitis* and *Staphylococcus aureus*. J. Bacteriol..

[169] Ajuebor J., McAuliffe O., O’Mahony J., Ross R.P., Hill C., Coffey A. (2016). Bacteriophage endolysins and their applications. Sci. Prog..

[170] Loeffler J.M., Nelson D., Fischetti V.A. (2001). Rapid killing of *Streptococcus pneumoniae* with a bacteriophage cell wall hydrolase. Science.

[171] Schuch R., Nelson D., Fischetti V.A. (2002). A bacteriolytic agent that detects and kills *Bacillus anthracis*. Nature.

[172] Pastagia M., Euler C., Chahales P., Fuentes-Duculan J., Krueger J.G., Fischetti V.A. (2011). A novel chimeric lysin shows superiority to mupirocin for skin decolonization of methicillin-resistant and -sensitive *Staphylococcus aureus* strains. Antimicrob. Agents Chemother..

[173] Becker S.C., Roach D.R., Chauhan V.S., Shen Y., Foster-Frey J., Powell A.M., Bauchan G., Lease R.A., Mohammadi H., Harty W.J. (2016). Triple-acting Lytic Enzyme Treatment of Drug-Resistant and Intracellular *Staphylococcus aureus*. Sci. Rep..

[174] Singh P.K., Donovan D.M., Kumar A. (2014). Intravitreal injection of the chimeric phage endolysin Ply187 protects mice from *Staphylococcus aureus* endophthalmitis. Antimicrob. Agents Chemother..

[175] Grishin A.V., Karyagina A.S., Vasina D.V., Vasina I.V., Gushchin V.A., Lunin V.G. (2020). Resistance to peptidoglycan-degrading enzymes. Crit. Rev. Microbiol..

[176] Ragland S.A., Criss A.K. (2017). From bacterial killing to immune modulation: Recent insights into the functions of lysozyme. PLoS Pathog..

[177] Kusuma C., Jadanova A., Chanturiya T., Kokai-Kun J.F. (2007). Lysostaphin-resistant variants of *Staphylococcus aureus* demonstrate reduced fitness in vitro and in vivo. Antimicrob. Agents Chemother..

[178] Stogios P.J., Savchenko A. (2020). Molecular mechanisms of vancomycin resistance. Protein Sci..

[179] Yang H., Yu J., Wei H. (2014). Engineered bacteriophage lysins as novel anti-infectives. Front. Microbiol..

[180] Rodríguez-Rubio L., Martínez B., Rodríguez A., Donovan D.M., García P. (2012). Enhanced staphylolytic activity of the *Staphylococcus aureus* bacteriophage vB_SauS-phiiPla88 HydH5 Virion-associated peptidoglycan hydrolase: Fusions, deletions, and synergy with LysH5. Appl. Environ. Microbiol..

[181] Fenton M., Ross P., Mcauliffe O., O’Mahony J., Coffey A. (2010). Recombinant bacteriophage lysins as antibacterials. Bioeng. Bugs.

[182] Rodríguez-Rubio L., Gutiérrez D., Donovan D.M., Martínez B., Rodríguez A., García P. (2016). Phage lytic proteins: Biotechnological applications beyond clinical antimicrobials. Crit. Rev. Biotechnol..

[183] São-José C., Costa A.R., Melo L.D.R. (2022). Editorial: Bacteriophages and Their Lytic Enzymes as Alternative Antibacterial Therapies in the Age of Antibiotic Resistance. Front. Microbiol..

[184] van der Ploeg J.R. (2008). Characterization of *Streptococcus gordonii* prophage PH15: Complete genome sequence and functional analysis of phage-encoded integrase and endolysin. Microbiology.

[185] Muharram M.M., Abulhamd A.T., Aldawsari M.F., Alqarni M.H., Labrou N.E. (2020). Development of staphylococcus enzybiotics: The ph28 gene of *Staphylococcus epidermidis* phage ph15 is a two-domain endolysin. Antibiotics.

[186] Yoong P., Schuch R., Nelson D., Fischetti V.A. (2004). Identification of a broadly active phage lytic enzyme with lethal activity against antibiotic-resistant *Enterococcus faecalis* and *Enterococcus faecium*. J. Bacteriol..

[187] Swift S.M., Rowley D.T., Young C., Franks A., Hyman P., Donovan D.M. (2016). The endolysin from the *Enterococcus faecalis* bacteriophage VD13 and conditions stimulating its lytic activity. FEMS Microbiol. Lett..

[188] Zimmer M., Vukov N., Scherer S., Loessner M.J. (2002). The murein hydrolase of the bacteriophage φ3626 dual lysis system is active against all tested *Clostridium perfringens* strains. Appl. Environ. Microbiol..

[189] Mayer M.J., Narbad A., Gasson M.J. (2008). Molecular characterization of a *Clostridium difficile* bacteriophage and its cloned biologically active endolysin. J. Bacteriol..

[190] Mayer M.J., Payne J., Gasson M.J., Narbad A. (2010). Genomic sequence and characterization of the virulent bacteriophage φCTP1 from *Clostridium tyrobutyricum* and heterologous expression of its endolysin. Appl. Environ. Microbiol..

[191] Griego A., Antinori B., Spitaleri A., Muzzolini I., Muzzioli S. (2023). Endolysin B as a new archetype in *M. tuberculosis* treatment. bioRxiv.

[192] Gaeng S., Scherer S., Neve H., Loessner M.J. (2000). Gene cloning and expression and secretion of *Listeria monocytogenes* bacteriophage-lyric enzymes in *Lactococcus lactis*. Appl. Environ. Microbiol..

[193] Gondil V.S., Harjai K., Chhibber S. (2020). Endolysins as emerging alternative therapeutic agents to counter drug-resistant infections. Int. J. Antimicrob. Agents.

[194] Rahman M.U., Wang W., Sun Q., Shah J.A., Li C., Sun Y., Li Y., Zhang B., Chen W., Wang S. (2021). Endolysin, a promising solution against antimicrobial resistance. Antibiotics.

[195] Lai M.J., Lin N.T., Hu A., Soo P.C., Chen L.K., Chen L.H., Chang K.C. (2011). Antibacterial activity of *Acinetobacter baumannii* phage ΦaB2 endolysin (LysAB2) against both Gram-positive and Gram-negative bacteria. Appl. Microbiol. Biotechnol..

[196] Deutsch S.M., Guezenec S., Piot M., Foster S., Lortal S. (2004). Mur-LH, the Broad-Spectrum Endolysin of *Lactobacillus helveticus* Temperate Bacteriophage φ-0303. Appl. Environ. Microbiol..

[197] Wang J., Liang S., Lu X., Xu Q., Zhu Y., Yu S., Zhang W., Liu S., Xie F. (2023). Bacteriophage endolysin Ply113 as a potent antibacterial agent against polymicrobial biofilms formed by enterococci and *Staphylococcus aureus*. Front. Microbiol..

[198] Kim S., Jin J.S., Choi Y.J., Kim J. (2020). LysSAP26, a New Recombinant Phage Endolysin with a Broad Spectrum Antibacterial Activity. Viruses.

[199] Fischetti V.A. (2010). Bacteriophage endolysins: A novel anti-infective to control Gram-positive pathogens. Int. J. Med. Microbiol..

[200] Schmelcher M., Loessner M.J. (2021). Bacteriophage endolysins—Extending their application to tissues and the bloodstream. Curr. Opin. Biotechnol..

[201] De Maesschalck V., Gutiérrez D., Paeshuyse J., Lavigne R., Briers Y. (2020). Advanced engineering of third-generation lysins and formulation strategies for clinical applications. Crit. Rev. Microbiol..

[202] Schaffner W., Melly M.A., Hash J.H., Koenig M.G. (1967). Lysostaphin: An enzymatic approach to staphylococcal disease. I. In vitro studies. Yale J. Biol. Med..

[203] King B.F., Biel M.L., Wilkinson B.J. (1980). Facile penetration of the *Staphylococcus aureus* capsule by lysostaphin. Infect. Immun..

[204] Bastos M.D.C.D.F., Coutinho B.G., Coelho M.L.V. (2010). Lysostaphin: A staphylococcal bacteriolysin with potential clinical applications. Pharmaceuticals.

[205] Jayakumar J., Kumar V.A., Biswas L., Biswas R. (2021). Therapeutic applications of lysostaphin against *Staphylococcus aureus*. J. Appl. Microbiol..

[206] Harrison E.F., Zygmunt W.A. (1967). Lysostaphin in experimental renal infections. J. Bacteriol..

[207] Schaffner W., Melly M.A., Koenig M.G. (1967). Lysostaphin: An enzymatic approach to staphylococcal disease. II. In vivo studies. Yale J. Biol. Med..

[208] Dixon R.E., Goodman J.S., Koenig M.G. (1968). Lysostaphin: An enzymatic approach to staphylococcal disease. 3. Combined lysostaphin-methicillin therapy of established staphylococcal abscesses in mice. Yale J. Biol. Med..

[209] Climo M.W., Patron R.L., Goldstein B.P., Archer G.L. (1998). Lysostaphin treatment of experimental methicillin-resistant *Staphylococcus aureus* aortic valve endocarditis. Antimicrob. Agents Chemother..

[210] Dajcs J.J., Thibodeaux B.A., Girgis D.O., Shaffer M.D., Delvisco S.M., O’Callaghan R.J. (2002). Immunity to lysostaphin and its therapeutic value for ocular MRSA infections in the rabbit. Investig. Ophthalmol. Vis. Sci..

[211] Placencia F.X., Kong L., Weisman L.E. (2009). Treatment of methicillin-resistant *Staphylococcus aureus* in neonatal mice: Lysostaphin versus vancomycin. Pediatr. Res..

[212] Walsh S., Shah A., Mond J. (2003). Improved pharmacokinetics and reduced antibody reactivity of lysostaphin conjugated to polyethylene glycol. Antimicrob. Agents Chemother..

[213] Shah A., Mond J., Walsh S. (2004). Lysostaphin-coated catheters eradicate *Staphylococccus aureus* challenge and block surface colonization. Antimicrob. Agents Chemother..

[214] Zhao H., Brooks S.A., Eszterhas S., Heim S., Li L., Xiong Y.Q., Fang Y., Kirsch J.R., Verma D., Bailey-Kellogg C. (2020). Globally deimmunized lysostaphin evades human immune surveillance and enables highly efficacious repeat dosing. Sci. Adv..

[215] Mądry A., Jendroszek A., Dubin G., Wladyka B. (2019). Production of Lysostaphin by Nonproprietary Method Utilizing a Promoter from Toxin–Antitoxin System. Mol. Biotechnol..

[216] Zhao H., Verma D., Li W., Choi Y., Ndong C., Fiering S.N., Bailey-Kellogg C., Griswold K.E. (2015). Depletion of T cell epitopes in lysostaphin mitigates anti-drug antibody response and enhances antibacterial efficacy in vivo. Chem. Biol..

[217] Kerr D.E., Plaut K., Bramley A.J., Williamson C.M., Lax A.J., Moore K., Wells K.D., Wall R.J. (2001). Lysostaphin expression in mammary glands confers protection against staphylococcal infection in transgenic mice. Nat. Biotechnol..

[218] Martin R.R., White A. (1968). The reacquisition of staphylococci by treated carriers: A demonstration of bacterial interference. J. Lab. Clin. Med..

[219] Quickel K.E., Selden R., Caldwell J.R., Nora N.F., Schaffner W. (1971). Efficacy and safety of topical lysostaphin treatment of persistent nasal carriage of *Staphylococcus aureus*. Appl. Microbiol..

[220] Stark F.R., Thornsvard C., Flannery E.P., Artenstein M.S. (1974). Systemic Lysostaphin in Man—Apparent Antimicrobial Activity in a Neutropenic Patient. N. Engl. J. Med..

